# Negative Association of Gulf War Illness Symptomatology with Predicted Binding Affinity of Anthrax Vaccine Antigen to Human Leukocyte (HLA) Class II Molecules

**DOI:** 10.3390/vaccines13010088

**Published:** 2025-01-18

**Authors:** Lisa M. James, Apostolos P. Georgopoulos

**Affiliations:** 1 The GWI and HLA Research Groups, Brain Sciences Center, Department of Veterans Affairs Health Care System, Minneapolis, MN 55417, USA; omega@umn.edu; 2 Department of Neuroscience, University of Minnesota Medical School, Minneapolis, MN 55455, USA; 3 Department of Psychiatry, University of Minnesota Medical School, Minneapolis, MN 55455, USA

**Keywords:** anthrax vaccination, Gulf War Illness, Human Leukocyte Antigen (HLA)

## Abstract

Background: Anthrax is a serious disease caused by *Bacillus anthracis* (*B. anthracis*) with a very high mortality when the spores of *B. anthracis* are inhaled (inhalational anthrax). Aerosolized *B. anthracis* spores can be used as a deadly bioweapon. Vaccination against anthrax is the only effective preventive measure and, hence, the anthrax vaccine was administered to United States (and other) troops during the 1990–91 Gulf War. However, the anthrax vaccine is not harmless, and the anthrax vaccination has been linked to the occurrence and severity of Gulf War Illness (GWI), a debilitating Chronic Multisymptom Illness (CMI). We hypothesized that this is partly due to the combination of two factors, namely (a) the cytotoxicity of the antigen (anthrax Protective Antigen, PA) contained in the vaccine, and (b) the Human Leukocyte Antigen (HLA) genotype of susceptible vaccinees, reducing their ability to make antibodies against the cytotoxic PA. Method: Here, we tested this hypothesis by determining the association between severity of GWI symptoms in 458 GW veterans and the overall strength of the binding affinity of the PA epitopes to the specific six Human Leukocyte Antigen (HLA) Class II alleles carried by each individual (two of each of the HLA-II genes: DPB1, DQB1, DRB1), responsible for initiating the process of antibody production in otherwise immunocompetent individuals, estimated in silico. Results: We found that the severity of GWI symptomatology was negatively and significantly correlated with the strength of the predicted binding affinity of PA peptides to HLA-II molecules (r=−0.356, p<0.001); the stronger the overall binding affinity, the weaker the symptoms. Since the binding of a peptide to an HLA-II molecule is the first and necessary step in initiating the production of antibodies, the findings above support our hypothesis that the severity of GWI symptomatology is partly due to a lack of HLA-II protection. Conclusions: Reduced HLA protection against the toxic anthrax vaccine may underlie GWI.

## 1. Introduction

Anthrax is a serious disease caused by *Bacillus anthracis* (*B. anthracis*) with a very high mortality when the spores of *B. anthracis* are inhaled (inhalational anthrax). Aerosolized *B. anthracis* spores can be used as a deadly bioweapon. Vaccination against anthrax is the only effective preventive measure and, hence, the anthrax vaccine was administered to United States (and other) troops during the 1990–91 Gulf War. However, the anthrax vaccine is not harmless, and the anthrax vaccination has been linked to the occurrence and severity of Gulf War Illness (GWI) [1,2], a debilitating Chronic Multisymptom Illness (CMI [3]). We hypothesized [2] that this is partly due to the combination of two factors, namely (a) the cytotoxicity of the antigen (anthrax Protective Antigen, PA) contained in the vaccine [4,5] , and (b) the Human Leukocyte Antigen (HLA) genotype of susceptible vaccinees [2], reducing their ability to make antibodies against the cytotoxic PA [6], as discussed in detail below. 

### 1.1. Anthrax

The spores of *B. anthracis* can enter the body via four routes, namely the lungs, the skin, the mouth, and by injection [7]. Consequently, there are four clinical forms of the disease, depending on the entry route: inhalational anthrax (lungs), cutaneous anthrax (skin), mouth (ingestional anthrax), and injection (injectional anthrax [8,9]. The mortality is highest for inhalational anthrax (up to 90%), lowest for cuteneous anthrax that can be treated successfully with antibiotics (penicillin, amoxicillin) [7], and intermediate for ingestional and injectional anthrax if antibiotic treatment is given very early [7,9]. The spores of *B. anthracis* can last for long periods of time and can be aerosolized and used as a bioweapon in bioterrorist attacks [10,11]. Hence, the need to protect individuals who may be at risk of exposure to anthrax is imperative. Such protection can be provided by the anthrax vaccine.

### 1.2. Anthrax Vaccine

The only known preventive measure for anthrax is the anthrax vaccine, hence the massive vaccination of United States (U.S.) armed forces personnel during the 1990-91 Gulf War [12]. The anthrax vaccine administered was a vaccine developed in 1963 from the “Strain V770-NPl-R, a nonencapsulated, nonproteolytic, and avirulent mutant of *B. anthracis*” [13] (p. 330); the main component of that vaccine was the “protective antigen, PA”, namely an 83 kDa protein (PA83), derived from a “sterile culture filtrate” [8] (p. 330). PA administration induces the production of neutralizing antibodies against *B. anthracis* [14,15,16] that are effective in protecting against the disease, as tested in animals [17,18]. However, the successful production of antibodies depends on the presence in the vaccine recipient of Human Leukocyte Antigen Class II (HLA-II) molecules [19] that can bind with the peptides (epitopes) of PA to form a stable peptide–HLA molecule complex (pHLA-II) which, in turn, activates CD4+ lymphocytes to initiate antibody production by B cells [20,21]. Now, every individual carries six classical HLA-II alleles (two of each one of three genes: DPB1, DQB1, DRB1) which make the corresponding HLA-II molecules to which the antigen peptides attach. The HLA system is the most polymorphic in the human genome, comprising thousands of alleles. This confers a major evolutionary, protective advantage to the human population as a whole against microbes but the likelihood that a specific individual will mount successful antibody production against a microbial antigen depends on the affinity of the six HLA-II that the individual carries to bind to antigen epitopes to form a stable pHLA-II complex. The successful formation of such a stable complex depends on the strength of the binding affinity between the peptide and the HLA-II molecule [22,23]; when successful, the pHLA complex possesses high sensitivity [24] and specificity [25]. In an ideal situation, when the HLA-II genotype of the vaccinee provides molecules that can bind with high affinity to antigen epitopes, antibodies will be made and protection conferred. On the other hand, if these molecules happen not to possess the requisite affinity to bind with the antigen epitopes, two consequences follow. First, antibodies are not made, or are produced in inadequate quantities, hence protection is inadequate; second, the vaccine antigen is not eliminated and may persist for a while, depending on the antigen and the condition of the host. In the case of the anthrax vaccine, both of these considerations are important for two main reasons. First, no (or only limited) protection is conferred against anthrax exposure, and second, a persistent anthrax vaccine antigen can be harmful to the host, possibly leading to chronic multisymptom illness, such as GWI, as discussed below.

### 1.3. Gulf War Illness (Chronic Multisymptom Illness)

Shortly after deployment in support of the 1990–91 Gulf War, veterans started complaining, mainly, of fatigue, pain, and neurocognitive dysfunction but also of gastrointestinal, respiratory, and dermatologic symptoms. This chronic, multisymptom constellation was acknowledged as an illness [26], called Gulf War Illness (GWI) and/or Chronic Multisymptom Illness (CMI) [3]. Overall, approximately 30% of ~600,000 U.S. GW veterans developed GWI.

### 1.4. Iraq and Afghanistan Wars

Remarkably, the very same GWI symptomatology was observed in veterans tested one year post-deployment to Iraq (Operation Iraqi Freedom, OIF) and Afghanistan (Operation Enduring Freedom, OEF) wars [27,28] which were twenty years after the 1990–91 Gulf War. More specifically, 49.5% of these veterans met the criteria for mild-to-moderate CMI and 10.8% met the criteria for severe CMI [10]. Moreover, “Over 90% of Veterans with chronic pain met criteria for CMI. CMI was not completely accounted for either by posttraumatic stress disorder or by predeployment levels of physical symptoms. Veterans with symptoms consistent with CMI reported significantly worse physical health function than Veterans who did not report symptoms consistent with CMI” [27] (p. 59). These are alarming statistics. Presumably, soldiers deployed to OIF/OEF received the anthrax vaccine, which was mandatory for troops deployed to the Middle East. Various environmental exposures unique to the 1990–91 Gulf War (e.g., exposure to burning pits) that have been blamed for GWI [29] did not occur in the OIF/OEF operations and, therefore, cannot account for the CMI symptoms associated with these wars.

### 1.5. Hypothesis of Persistent Anthrax Vaccine Antigen in GWI

In 2016, we reported on the lack of HLA protection in GWI [6] and hypothesized that this may be at the core of GWI. More specifically, we hypothesized that GWI is due to pathogenic antigen(s) to which all GW veterans were exposed and which were eliminated by the healthy GW veterans but could not be eliminated by other veterans, hence their persistence in the body of the latter, leading to the development of GWI. We further refined this hypothesis to focus on the cytotoxic PA anthrax vaccine antigen [5,30] and proposed that GWI is partially due to the persistent presence of PA in veterans suffering from GWI [31], a persistence more specifically due to reduced ability by the afflicted veterans to produce antibodies against PA. This hypothesis was supported by the results of a recent epidemiological study [2] which found significant associations among anthrax vaccination, risk of developing GWI, and HLA genotype that helps eliminate foreign antigens (Human Leukocyte Antigen, HLA).

### 1.6. Human Leukocyte Antigen (HLA)

Briefly, HLA is the most polymorphic system in the human genome and is the genetic core of adaptive immunity [20,21]. It consists of two classes of genes, namely Class I and Class II. HLA-II molecules are instrumental in initiating the production of antibodies to foreign antigens. These molecules are expressed on antigen-presenting cells, bind and present endocytosed exogenous antigen epitopes to CD4+ T cells to stimulate antibody production and adaptive immunity. The crucial step in this process is the binding with high affinity of antigen epitopes to HLA-II molecules [20,22,23].

### 1.7. HLA, Anthrax Vaccine, and GWI

The objective of the anthrax (and any other) vaccination is to enable the production of neutralizing antibodies specific to the vaccine’s antigen, derived from a targeted pathogen. In the case of future exposure to the pathogen, it is expected that these antibodies will “neutralize” the offending pathogen and thus prevent infection. With respect to the anthrax vaccine, studies in animals and human subjects [1,14,15,16,17,18,19,32] have documented the presence of such neutralizing antibodies and confirmed the protection conferred by the vaccine against anthrax exposure in vaccinated animals. However, the degree of protection strictly depends on the presence of sufficient quantities of neutralizing antibodies, which, in turn, depends on the presence in the host of HLA Class II alleles capable of producing the requisite antibodies, assuming immunocompetence otherwise. However, substantial variation in such antibody production was observed indicating that protection against exposure can vary, depending on the vaccinee’s HLA genotype [33]. In addition to lower protection against future exposure to anthrax, a reduced ability of the vaccinee’s HLA to make anti-PA antibodies means that the PA anthrax vaccine antigen administered will be eliminated only to the degree allowed by the amount of antibodies produced. In this case, PA will stay in the body as a “persistent antigen” [34] causing damage in various organs, given its known cytotoxicity [4,5]. Indeed, this is our hypothesized mechanism by which the persistent anthrax vaccine antigen PA could contribute to the development of GWI.

### 1.8. This Study: Testing the GWI–Lack of HLA-II Protection Hypothesis

Here, we directly tested the hypothesis above by (a) determining the severity of GWI symptomatology in 458 GW veterans, (b) characterizing the HLA-II genotype of each veteran, (c) estimating the strength of predicted binding affinity (PBA) of peptides (epitopes) of the anthrax vaccine antigen (PA) to the specific HLA-II molecules present in each veteran, and (d) correlating the severity of GWI symptomatology to the strength of PBA.

## 2. Materials and Methods

### 2.1. Participants

A total of 458 U.S. Gulf War veterans (age 56.27 ± 0.45 y, mean ± SEM; 397 men, 61 women) participated in the study as paid volunteers. The study was approved by the Minneapolis VA Medical Center Institutional Review Board and informed consent was obtained from all participants, according to the Declaration of Helsinki.

### 2.2. GWI Status and Severity

GWI symptom severity was assessed in the following 6 domains: fatigue, pain, neurological/cognitive/mood, respiratory, gastrointestinal, and dermatologic [3]. Only symptoms of at least moderate severity, that began during or after the Gulf War and lasted > 6 months counted towards a particular symptom domain. The overall GWI symptom severity score was the average of severity scores in the 6 symptom domains above.

### 2.3. B. anthracis Protective Antigen (PA)

The amino acid (AA) sequence of anthrax vaccine antigen (AVA) PA83, the active ingredient of the anthrax vaccine administered to Gulf War veterans, was retrieved from the Uniprot website (https://www.uniprot.org/uniprotkb/P13423/entry accessed on 27 April 2023). The PA consists of 764 amino acids (AA) whose sequence is given in Table S1 (Supplementary Material).

### 2.4. Human Leukocyte Antigen (HLA) Genotyping

DNA isolation was carried out from whole blood or saliva samples using commercially available kits (blood: ArchivePure cat. 2300730 from 5Prime distributed by Fisher Scientific or VWR; saliva: Oragene-Discover cat.OGR-500 coupled with prepIT purifier reagent cat.PT-L2P/DNA Genotek Inc. Ottawa, ON, Canada). The purified DNA samples were sent to Histogenetics ((http://www.histogenetics.com/,) accessed on 3 January 2024) for high-resolution (2-field) HLA Sequence-based Typing (SBT;). Histogenetics sequencing DNA templates are produced by locus- and group-specific amplifications that include exon 2 and 3 for class I (A, B, C) and exon 2 for class II (DRB1, DQB1, and DPB1) and reported as Antigen Recognition Site (ARS) alleles as per ASHI recommendation [35]. In our population, we identified 93 HLA-II alleles, shown in Table S2.

### 2.5. PA-HLA-II Binding Affinities: In Silico Estimation of Predicted Binding Affinity Between PA Peptides and HLA-II Allele Motifs

**Figure 1:**
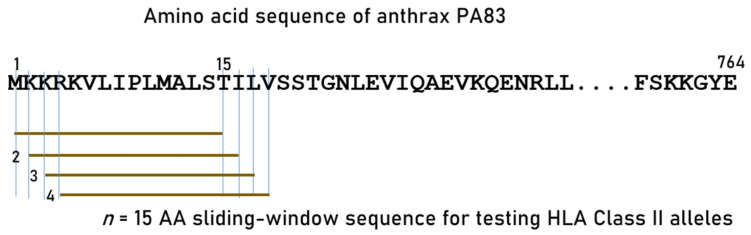
Schematic diagram to illustrate the sliding window approach for estimating in silico predicted binding affinities of 15-mers to HLA-II molecules.

We tested exhaustively all linear 15-AA length subsequences (epitopes; 15-mer) of PA using a sliding window approach [2] to partition the whole AA sequence of PA (number of 15-mer epitopes = 764 AA − 15 AA = 750; Figure 1). Predicted binding affinities of each PA epitope to each HLA Class II molecule carried by the Gulf War veterans were obtained for PA epitopes using the Immune Epitope Database (IEDB) NetMHCpan (version 4.1) tool [36,37]. For each pair of peptide–HLA molecule (pHLA-II) tested, this tool gives, as an output, the ic50 of the predicted binding affinity (PBA); the smaller the ic50, the stronger the binding affinity; an ic50 value of ≤50 nm is regarded strong [38]. We called these strong binders “hits” and assigned them a value of 1; PBA values > 50 nM were assigned the value 0. Thus, for a peptide i and allele k, we have

(1) $$If\;ic50\left(p_{i}{HLA}_{k}\right)\leq 50\;nM,\;then\;H_{i}^{k}=1\;(\mathrm{HIT})$$

(2) $$If\;ic50\left(p_{i}{HLA}_{k}\right)>50\;nM,\;then\;H_{i}^{k}=0$$

Given that 750 15-mer peptides were tested for each HLA allele, there were 750 PBAs per allele, each of which could be a HIT or not. For an allele k, the sum of all 750 $H_{i}^{k}$ values was computed as an estimate of the overall strength of binding capacity of the allele to PA. Let i by a 15-AA peptide (N = 750) and k be a specific allele. Then, we have

(3) $${aH}_{k}=\sum_{i}^{i=1,750}H_{i}^{k}$$

Finally, given that a given individual carries 6 alleles (2 of each 3 HLA-II genes: DPB1, DQB1, DRB1), the overall strength of pHLA-II binding capacity for a subject m can be computed as the sum of $aH_{k}$ across the 6 alleles carried by the subject:

(4) $$sH_{m}=\sum_{k}^{k=1,6}{aH}_{k}$$

This is an estimate of the overall ability of the subject to engage CD4+ cells for antibody production.

### 2.6. Data Analysis

Standard statistical methods were employed to analyze the data, including descriptive statistics, *t*-tests, correlation, partial correlation, and nonparametric analyses. The IBM-SPSS statistical package (version 29) was used for all statistical analyses. All correlation coefficients are Pearson correlations and all reported *p*-values are 2-tailed.

## 3. Results

### 3.1. Hits

**Figure 2:**
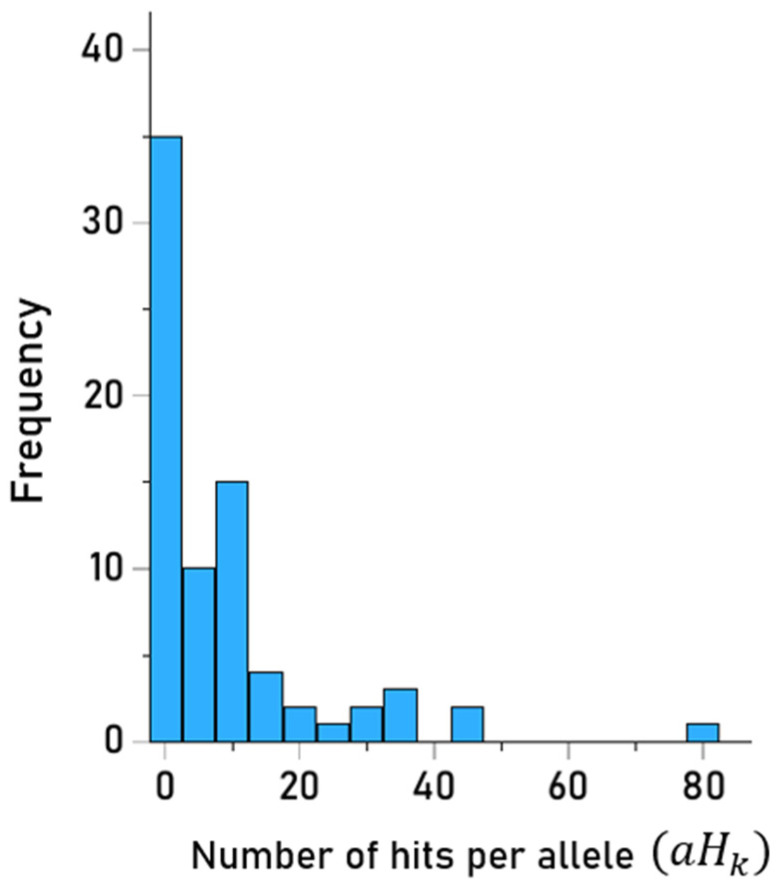
Frequency distribution of hits of HLA-II alleles (N = 75 DPB1 and DRB1 alleles).

**Figure 3:**
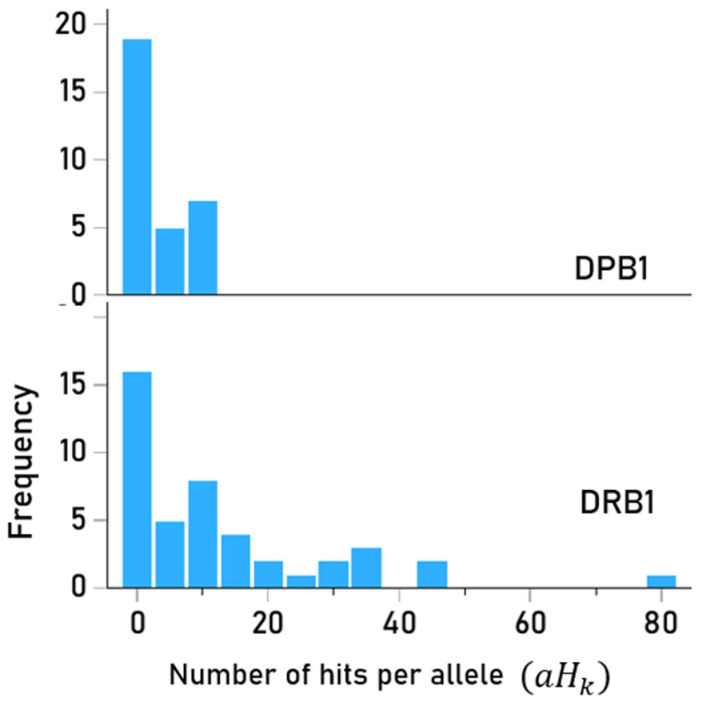
AFrequency distributions of hits for DPB1 (N = 31) and DRB1 (N = 44) alleles.

There was a total of 661 hits; their values across the 93 HLA-II alleles tested are shown in Table S3. It can be seen that alleles of the DQB1 gene did not have any hit. The frequency distribution of hits per allele is shown in Figure 2. With respect to specific genes, the frequency distributions of hits for DPB1 (N = 31) and DRB1 (N = 44) genes are shown in Figure 3.

### 3.2. Association of GWI Symptom Severity and $sH_{m}$

**Figure 4:**
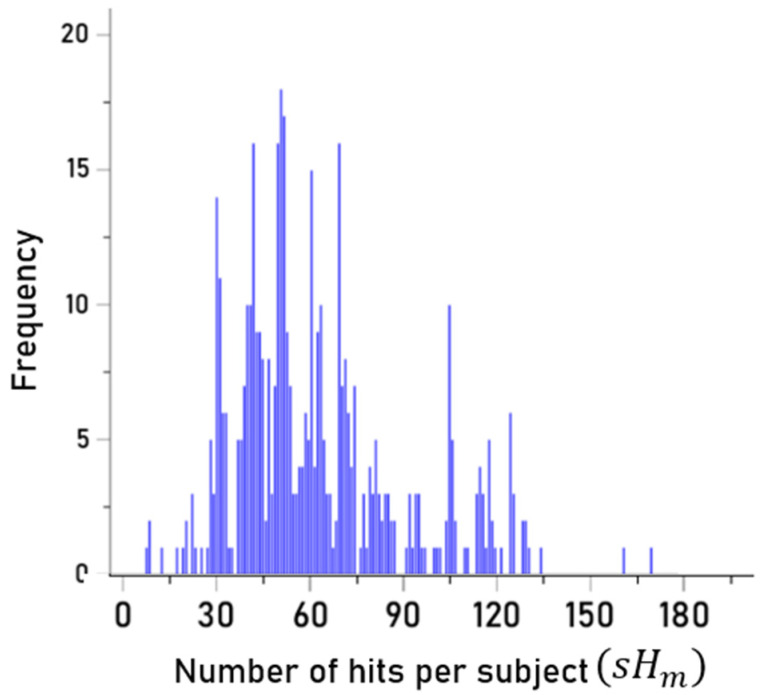
Frequency distribution of hits per subject (N = 458 participants).

**Figure 5:**
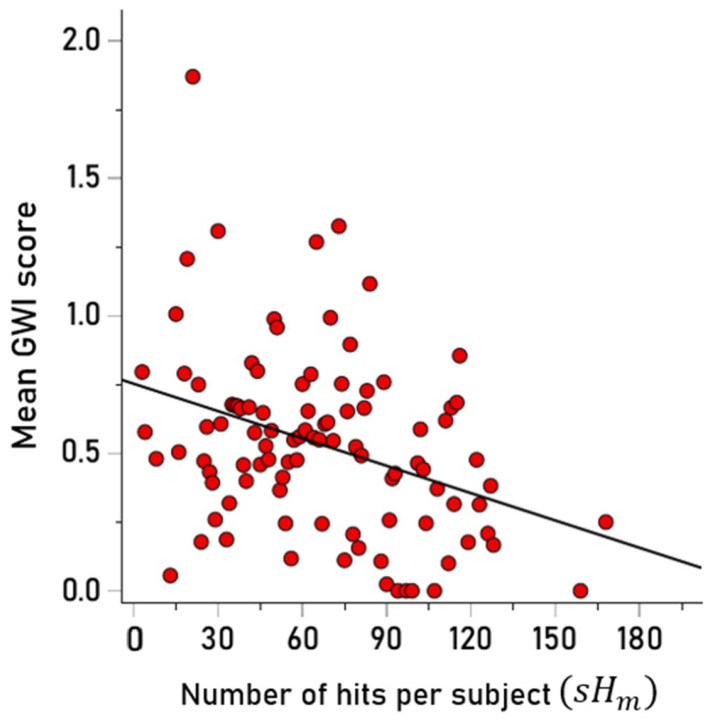
The mean GWI score per $sH_{m}$ is plotted against $sH_{m}$. N = 98 distinct $sH_{m}$ values. See text for details.

Since $aH_{k}$ is an integer (the counts of hits per HLA-II allele), $sH_{m}$ is also an integer, which means that each individual had a $sH_{m}$ value, i.e., the sum of all hits of the alleles the individual carried. Thus there were 458 $sH_{m}$ values, one per participant; their frequency distribution is shown in Figure 4. These 458 $sH_{m}$ values consisted of 98 distinct values, reflecting the fact that several participants had the same $sH_{m}$. The association between $sH_{m}$ and GWI symptom severity was investigated by computing the average GWI symptom severity score for each one of the 98 distinct $sH_{m}$ values above and then regressed against the corresponding $sH_{m}$ values. We found that GWI severity scores were negatively and significantly associated with $sH_{m}$ (Figure 5) ( r=−0.356, p<0.001), which means that GWI severity decreased as the number of hits increased; this relation was independent of age (partial correlation, controlling for age, between GWI score and $sH_{m}$ $r_{p}=-0.376,\;p<0.001$). Overall, these findings document a protective effect of HLA hits on GWI symptom severity. Finally, we compared the number of hits between the groups with and without GWI symptoms (GWI score > 0 vs. GWI score = 0) with respect to 18 alleles that were absent in the GWI group in our previous study [2] and found that, indeed, the number of hits were significantly higher in the non-GWI group (*p* = 0.018, Wilcoxon Signed Rank test), further confirming the protective role of these alleles.

### 3.3. Candidate Vaccine Epitopes

**Figure 6:**
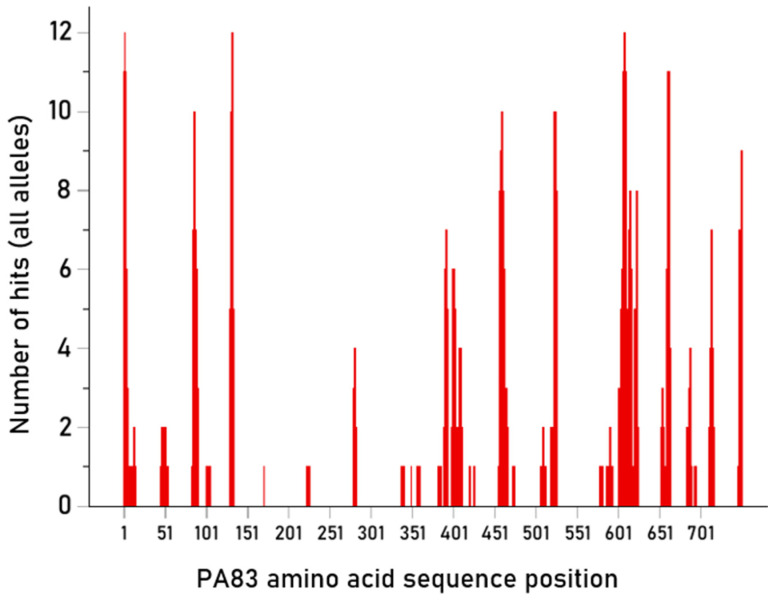
Location of hits (N = 661) along the PA amino acid sequence.

**Figure 7:**
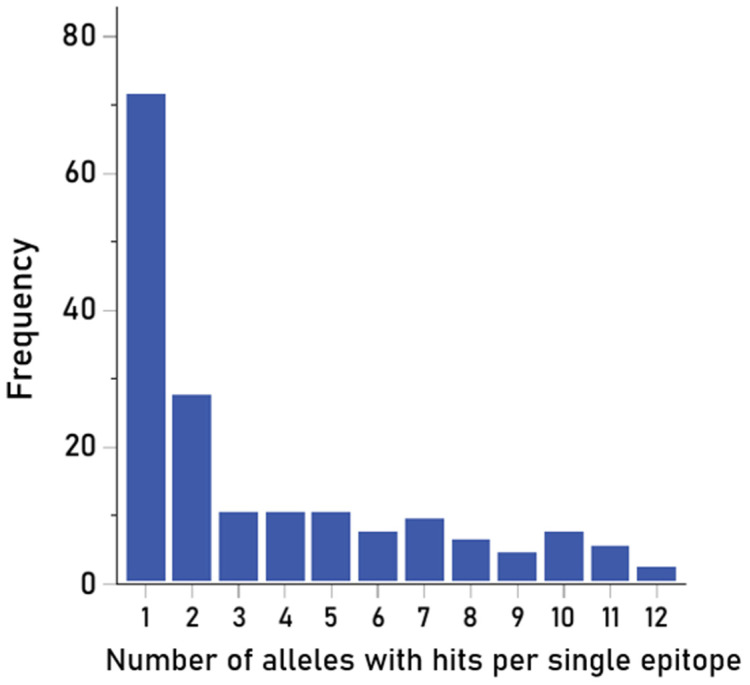
Frequency distribution of the number of hits per epitope.

The purpose of this analysis was to identify PA 15-mer epitopes with high PBA over all alleles tested. Such epitopes would be good candidates as constituents of peptide, multiepitope-based, anti-anthrax PA vaccines. The number of hits for each of the 750 15-mer epitopes tested is given in Table S4 (together with their mean ic50 values) and shown in Figure 6 along the PA amino acid sequence. Altogether, there were 180/750 (24%) 15-mer epitopes with hits, i.e., strong binding affinities to HLA-II molecules; these epitopes would have a good chance to engage CD4+ lymphocytes to initiate the process of antibody production. An additional consideration with respect to the practical value of specific epitopes in vaccine development concerns the number of strong HLA-II binders for a particular epitope. This number ranged from 1 to 12, as shown in Table S5 and depicted in Figure 7; epitopes that can bind strongly to more HLA-II molecules would be better candidates for multi-epitope anti-anthrax vaccine development.

More specifically, there were three epitopes with strong PBA to 12 HLA-II molecules, six with strong binding to 11 HLA-II molecules, and eight with strong binding to 10 HLA-II molecules. Overall, these epitopes bound strongly to 40/93 (43%) discreet HLA-II molecules, ensuring a good chance of CD4+ engagement across different individuals.

## 4. Discussion

### 4.1. GWI, Anthrax Vaccination and HLA

This study tested the hypothesis that GWI symptom severity is partly due to reduced protection conferred by the HLA-II system, necessary for antibody production. Specifically, we estimated in silico the predicted binding affinity of anthrax vaccine antigen (PA) epitopes to the HLA-II molecules present in a large sample of Gulf War veterans and documented a highly significant negative association between the strength of binding affinity of PA epitopes to HLA-II molecules and GWI symptom severity. These findings indicate that veterans possessing molecules of HLA alleles that are capable of binding PA epitopes with high affinity are not only more likely to develop antibodies against anthrax, the very purpose of vaccination, but also have enhanced protection against developing GWI symptoms. Conversely, veterans carrying fewer HLA-II molecules with high-affinity binding to PA epitopes would exhibit greater GWI symptom severity. Since individuals possessing HLA-II molecules with weak binding affinity for anthrax vaccine antigen epitopes are relatively less capable of antibody production against anthrax vaccine antigen, they will be afforded less protection in the event of anthrax exposure and are more likely to experience GWI, raising additional questions about the suitability of universal anthrax vaccination for U.S. veterans [1].

Previous research documented that the presence of certain Class II alleles, but no Class I alleles, discriminated between healthy Gulf War veterans and those with GWI such that healthy veterans possessed certain Class II alleles that were absent or significantly less frequent in those with GWI [6]. Subsequent research found that of the 69 HLA Class I alleles investigated, none were characterized by the combination of high-affinity binding to PA epitopes and high immunogenicity to anthrax vaccine antigen whereas several HLA Class II alleles were [2]. The unique effect of Class II HLA on GWI reported now in several studies suggests that protection against GWI or lack thereof, at least with regard to anthrax vaccination, sits squarely with antibody production, the very role of Class II HLA. Since high-affinity binding between a peptide and an HLA-II molecule (pHLA-II complex) is necessary for antibody production, only those individuals possessing HLA-II molecules capable of binding with sufficient affinity with PA epitopes will be able to mount an antibody response; here, strong PA binding was limited to DPB1 and DRB1 alleles (Figure 3). Since each individual possesses two DPB1 and two DRB1 alleles whose molecules vary with respect to the strength of binding with epitopes of the anthrax vaccine antigen, there is wide variability across individuals concerning the relative likelihood of high-affinity pHLA binding hits (‘hits’), ranging here from a total of three hits in some individuals to 168 hits in others (Figure 4). In the current sample, a number of veterans had few hits $\left(sH_{m}\right)$, meaning that those individuals would be less protected against anthrax exposure due to insufficient ability to make antibodies against anthrax vaccine antigen PA, and would thus be at a greater risk for developing GWI symptoms. Furthermore, in the absence of high-affinity binding, the vaccine antigen epitopes may persist [34]. Since the vaccine antigen is cytotoxic [4,5], the persistence of those antigens may cause damage in various organs. Indeed, we proposed that circulating persistent anthrax vaccine contributes to the array of GWI symptoms that span several organ systems and continue to impact roughly one-third of veterans decades after the Gulf War [31]. Crucial evidence for the presence of PA in the serum of veterans with GWI has been provided by the results of studies documenting the beneficial effect of specific monoclonal and/or polyclonal anti-PA antibodies in reducing substantially the toxic effects of PA in neural cultures [5,30]. More specifically, the addition of serum from GWI patients to neuroblastoma N2A cultures induced decreased neurite spreading and cell death, both of which were partly reversed by the addition of anti-PA antibodies [30]. Moreover, the disruption of various cellular processes caused by PA on the integrity of cell membrane, cytoskeleton and mitochondria was reversed by the addition of anti-PA antibodies [5].

### 4.2. Binding Affinity of PA Epitopes to HLA-II Molecules

The overall protection afforded by anthrax vaccination is determined by the ability of the combination of alleles each individual possesses to facilitate antibody production. That is, if all of an individual’s alleles bind weakly with the anthrax vaccine antigen PA, that individual will be minimally protected whereas possessing one or more alleles that bind strongly with the anthrax vaccine PA will increase protection such that the more antibodies produced, the better. In this case, several HLA-II molecules (15/31 (48%) DPB1, 18/18 (100%) DQB1, 13/44 (30%) DRB1) did not have even a single hit of strong binding affinity (ic50 ≤ 50 nM) with any of the 750 PA epitopes. On the other hand, some alleles were characterized by numerous hits. For example, the DRB1*01:01 HLA molecule had 78 strong binding hits with PA epitopes, the most for any of the alleles investigated here, and DRB1*13:02 had 43 hits. Notably, these were two of the alleles that had previously been shown to discriminate between healthy Gulf War veterans and those with GWI [6]. The present findings suggest that those alleles may promote health by enhanced antibody production.

### 4.3. Implications for Peptide-Based, Multi-Epitope Anthrax Vaccine Design

**Figure 8:**
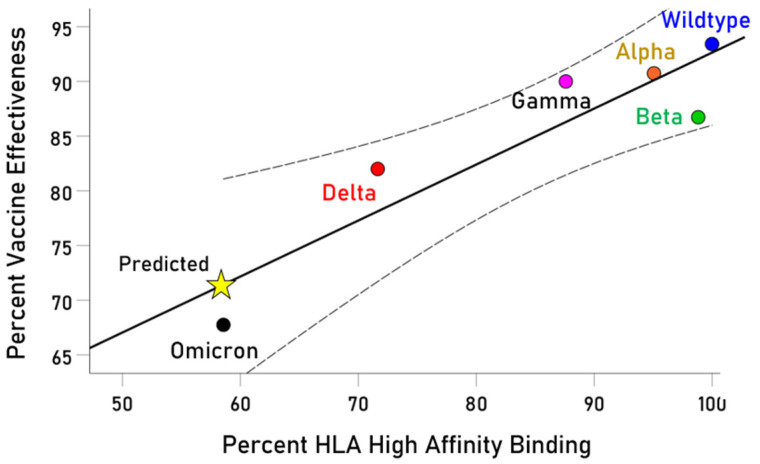
Dependence of COVID-19 vaccine effectiveness on HLA high-binding affinity to SARS-CoV-2 epitopes. Dashed lines are 95% mean confidence intervals. (From [45] http://creativecommons.org/licenses/by/4.0)/ The yellow star indicates the prediction for the Omicron variant.

The effectiveness of a vaccine in protecting against a disease upon exposure depends on the presence of effective antibodies against the offending microorganism [39,40]. Now, the first necessary step in the process of initiating antibody production is the formation of a stable pHLA-II complex [20,21,22,23], which defines most exclusively T cell epitopes [22,23,41,42]. It follows that peptide binding to an HLA-II molecule is the prerequisite for CD4+ T cell recognition for initiating antibody production by B cells in otherwise immunocompetent individuals and ensuing vaccine effectiveness. These considerations imply that the effectiveness of a vaccine will depend on the strength of the binding affinity of the epitopes used in the vaccine to HLA-II molecules. In a clinical setting of evaluating vaccine effectiveness, the same vaccine is administered to many individuals, which means that the same antigen epitopes are presented to the many different HLA-II molecules carried by the individuals in the vaccinated population. Therefore, the hypothesized dependence of clinical vaccine effectiveness on the strength of peptide (epitope)-HLA-II molecules can be tested by assessing the dependence of the average outcome of clinical studies of vaccine effectiveness on the average strength of peptide–HLA-II binding affinity. Of these two crucial measures, the outcome of clinical studies is known directly, whereas the pHLA-II binding affinity can be estimated in silico using well-established, reliable prediction algorithms [36,37,43,44]. Indeed, we successfully tested this hypothesis in the context of vaccines made for five variants of the SARS-CoV-2 virus (Wildtype, Alpha, Beta, Gamma, Delta, Omicron) and 56 common HLA-II alleles with frequencies ≥ 0.01 [45]. We found that the average clinical vaccine effectiveness was highly correlated with the average high in silico predicted binding affinity of the SARS-CoV-2 variant spike glycoprotein epitopes to the HLA-II molecules tested ( r=0.910, p=0.013; N = six SARS-CoV-2 variants; Figure 8). This finding validates the in silico approach in identifying antigen epitopes with predicted high binding affinity to HLA-II molecules as prospectively good candidates for vaccine development. This epitope-based approach is in keeping with other approaches in designing multiepitope-based immunogens for anthrax [46,47] and other diseases [48,49,50,51,52,53,54]. Finally, it should be pointed out that the high-affinity binding of a peptide antigen (Ag) to an HLA-II molecule is the necessary first step in initiating antibody production, as was stressed by Mahanty et al. [55]: “The central issue of peptide immunogenicity is Ag presentation” [55] (p. 3). Although the successful production of antibodies ultimately depends on the immunocompetence in all intermediate steps following antigen presentation to CD4+ T lymphocytes until the antibody production by B cells, the high-affinity binding of the potential immunogenic peptide to an HLA-II molecule is a necessary prerequisite. Studies on peptide immunogenicity typically take for granted successful antigen presentation but that success depends on the strength of binding affinity of the Ag to the HLA-II molecule, a condition that cannot (and should not) be taken for granted. Hence, the identification in this study of PA peptides with high binding affinities to HLA-II molecules is important as the first step in selecting candidate peptides for anthrax vaccine development. Further evaluation of these peptides with respect to their immunogenicity remains to be investigated.

### 4.4. Conclusions

The current findings, demonstrating a robust negative association between HLA-anthrax vaccine PA binding and GWI symptom severity, strongly support the hypothesized role of reduced antibody production against anthrax vaccine PA in GWI that most probably underlies the findings supporting anthrax antigen persistence in GWI [30], in the broader context of antigen persistence in other diseases [34,56,57,58,59]. Although this does not preclude other contributory factors in GWI [29], it does strongly point to anthrax vaccination in the absence of relevant strongly binding Class II HLA alleles in GWI symptom severity, presumably due to cytotoxic effects of persistent anthrax vaccine antigen [4,5]. Since the anthrax vaccine continues to be routinely administered to U.S. veterans, the present findings add support to calls for greater examination of immunogenetic risks and potential cytotoxic effects associated with the current anthrax vaccination program [1] in order to mitigate potentially harmful effects of anthrax vaccination on our armed forces.

## Data Availability

The raw data supporting the conclusions of this article will be made available by the authors upon request.

## Supplementary Materials

The following supporting information can be downloaded at: https://www.mdpi.com/article/10.3390/vaccines13010088/s1. **Table S1.** Amino acid sequences of anthrax protective antigen. **Table S2**. HLA Class II alleles used. **Table S3.** Counts (N) of hits (strongly binding peptide-HLA-II complexes:pHLA-II; ic50 ≤ 50 nM) for the alleles tested. **Table S4.** Sequence, location along the PA aminoacid sequence, mean ic50 and N of strongly binding peptide-HLA-II complexes (hits). **Table S5.** Epitope sequences with multiple allele hits, presented in alphabetical order of the sequence. Start and End denote the location of the epitope along the PA amino acid sequence (Table S1).
