# Supplementary material for: Negative Association of Gulf War Illness Symptomatology with Predicted Binding Affinity of Anthrax Vaccine Antigen to Human Leukocyte (HLA) Class II Molecules
Source: Vaccines (Basel). 2025 Jan 18;13(1):88. doi: 10.3390/vaccines13010088 (PMC11768865; doi:10.3390/vaccines13010088)
Supplement: Supplementary file 1 [file vaccines-13-00088-s001.zip › vaccines-3403289-supplementary.pdf]

Table S1. Amino acid sequences of anthrax protective antigen.

Labels are from https://www.uniprot.org/uniprotkb/P13423/entry

P13423 · PAG_BACAN	Protective antigen (Bacillus anthracis)	764 AA

MKKRKVLIPLMALSTILVSSTGNLEVIQAEVKQENRLLNESESSSQGLLGYYFSDLNFQAPMVVTSSTTGDLSIPSSEL
ENIPSENQYFQSAIWSGFIKVKKSDEYTFATSADNHVTMWVDDQEVINKASNSNKIRLEKGRLYQIKIQYQRENPTEKG
LDFKLYWTDSQNKKEVISSDNLQLPELKQKSSNSRKKRSTSAGPTVPDRDNDGIPDSLEVEGYTVDVKNKRTFLSPWIS
NIHEKKGLTKYKSSPEKWSTASDPYSDFEKVTGRIDKNVSPEARHPLVAAYPIVHVDMENIILSKNEDQSTQNTDSQTR
TISKNTSTSRTHTSEVHGNAEVHASFFDIGGSVSAGFSNSNSSTVAIDHSLSLAGERTWAETMGLNTADTARLNANIRY
VNTGTAPIYNVLPTTSLVLGKNQTLATIKAKENQLSQILAPNNYYPSKNLAPIALNAQDDFSSTPITMNYNQFLELEKT
KQLRLDTDQVYGNIATYNFENGRVRVDTGSNWSEVLPQIQETTARIIFNGKDLNLVERRIAAVNPSDPLETTKPDMTLK
EALKIAFGFNEPNGNLQYQGKDITEFDFNFDQQTSQNIKNQLAELNATNIYTVLDKIKLNAKMNILIRDKRFHYDRNNI
AVGADESVVKEAHREVINSSTEGLLLNIDKDIRKILSGYIVEIEDTEGLKEVINDRYDMLNISSLRQDGKTFIDFKKYN
DKLPLYISNPNYKVNVYAVTKENTIINPSENGDTSTNGIKKILIFSKKGYEIG

Table S2. HLA Class II alleles used.

DPB1	DQB1	DRB1
DPB1*01:01	DQB1*02:01	DRB1*01:01
DPB1*02:01	DQB1*02:02	DRB1*01:02
DPB1*02:02	DQB1*03:01	DRB1*01:03
DPB1*03:01	DQB1*03:02	DRB1*03:01
DPB1*04:01	DQB1*03:03	DRB1*03:02
DPB1-04:02	DQB1*03:04	DRB1*04:01
DPB1-05:01	DQB1*03:05	DRB1*04:02
DPB1-06:01	DQB1*03:12	DRB1*04:03
DPB1-09:01	DQB1*03:19	DRB1*04:04
DPB1-10:01	DQB1*04:02	DRB1*04:05
DPB1-105:01	DQB1*05:01	DRB1*04:06
DPB1-11:01	DQB1*05:02	DRB1*04:07
DPB1*124:01	DQB1*05:03	DRB1*04:08
DPB1*126:01	DQB1*06:01	DRB1*04:11
DPB1-13:01	DQB1*06:02	DRB1*07:01
DPB1*131:01	DQB1*06:03	DRB1*08:01
DPB1*14:01	DQB1*06:04	DRB1*08:02
DPB1*15:01	DQB1*06:09	DRB1*08:03
DPB1*16:01		DRB1*08:04
DPB1*17:01		DRB1*08:11
DPB1*18:01		DRB1*09:01
DPB1*19:01		DRB1*10:01
DPB1*20:01		DRB1*11:01
DPB1*23:01		DRB1*11:02
DPB1*29:01		DRB1*11:03
DPB1*35:01		DRB1*11:04
DPB1*40:01		DRB1*12:01
DPB1*46:01		DRB1*13:01

DPB1*50:01		DRB1*13:02
DPB1*59:01		DRB1*13:03
DPB1*92:01		DRB1*13:04
		DRB1*13:05
		DRB1*13:06
		DRB1*13:12
		DRB1*14:01
		DRB1*14:02
		DRB1*14:04
		DRB1*14:06
		DRB1*14:54
		DRB1*15:01
		DRB1*15:02
		DRB1*15:03
		DRB1*16:01
		DRB1*16:02

Table S3. Counts (N) of hits (strongly binding peptide-HLA-II complexes:pHLA-II; ic50 ≤ 50 nM) for the alleles tested.

Allele	N
DPB1*01:01	2
DPB1*02:01	12
DPB1*02:02	12
DPB1*03:01	3
DPB1*04:01	11
DPB1-04:02	0
DPB1-05:01	0
DPB1-06:01	0
DPB1-09:01	0
DPB1-10:01	0
DPB1-105:01	0
DPB1-11:01	0
DPB1*124:01	3
DPB1*126:01	11
DPB1-13:01	0
DPB1*131:01	0
DPB1*14:01	2
DPB1*15:01	8
DPB1*16:01	0
DPB1*17:01	0
DPB1*18:01	0
DPB1*19:01	0
DPB1*20:01	3
DPB1*23:01	11
DPB1*29:01	0
DPB1*35:01	1

DPB1*40:01	4
DPB1*46:01	12
DPB1*50:01	2
DPB1*59:01	0
DPB1*92:01	3
DQB1*02:01	0
DQB1*02:02	0
DQB1*03:01	0
DQB1*03:02	0
DQB1*03:03	0
DQB1*03:04	0
DQB1*03:05	0
DQB1*03:12	0
DQB1*03:19	0
DQB1*04:02	0
DQB1*05:01	0
DQB1*05:02	0
DQB1*05:03	0
DQB1*06:01	0
DQB1*06:02	0
DQB1*06:03	0
DQB1*06:04	0
DQB1*06:09	0
DRB1*01:01	78
DRB1*01:02	15
DRB1*01:03	0
DRB1*03:01	22
DRB1*03:02	0
DRB1*04:01	12
DRB1*04:02	0
DRB1*04:03	0
DRB1*04:04	11
DRB1*04:05	3
DRB1*04:06	0
DRB1*04:07	0
DRB1*04:08	12
DRB1*04:11	0
DRB1*07:01	26
DRB1*08:01	0
DRB1*08:02	0
DRB1*08:03	0
DRB1*08:04	8
DRB1*08:11	0
DRB1*09:01	9
DRB1*10:01	44
DRB1*11:01	12

DRB1*11:02	33
DRB1*11:03	30
DRB1*11:04	30
DRB1*12:01	2
DRB1*13:01	33
DRB1*13:02	43
DRB1*13:03	2
DRB1*13:04	33
DRB1*13:05	12
DRB1*13:06	22
DRB1*13:12	0
DRB1*14:01	4
DRB1*14:02	8
DRB1*14:04	0
DRB1*14:06	16
DRB1*14:54	4
DRB1*15:01	13
DRB1*15:02	2
DRB1*15:03	6
DRB1*16:01	3
DRB1*16:02	13

Table S4. Sequence, location along the PA aminoacid sequence, mean ic50 and N of strongly binding peptide-HLA-II complexes (hits).

Index	Peptide	Start	End	Mean ic50 (nM)	N
1	MKKRKVLIPLMALST	1	15	28.451	11
2	KKRKVLIPLMALSTI	2	16	27.732	12
3	KRKVLIPLMALSTIL	3	17	33.113	11
4	RKVLIPLMALSTILV	4	18	28.270	6
5	KVLIPLMALSTILVS	5	19	22.180	3
6	VLIPLMALSTILVSS	6	20	32.470	2
7	LIPLMALSTILVSST	7	21	21.830	1
8	IPLMALSTILVSSTG	8	22	22.330	1
9	PLMALSTILVSSTGN	9	23	26.850	1
10	ALSTILVSSTGNLEV	12	26	33.760	1
11	LSTILVSSTGNLEVI	13	27	36.675	2
12	STILVSSTGNLEVIQ	14	28	29.300	1
13	TILVSSTGNLEVIQA	15	29	45.580	1
14	QGLLGYYFSDLNFQA	46	60	47.700	1
15	GLLGYYFSDLNFQAP	47	61	33.570	2
16	LLGYYFSDLNFQAPM	48	62	25.895	2
17	LGYYFSDLNFQAPMV	49	63	23.350	2
18	GYYFSDLNFQAPMVV	50	64	31.675	2
19	YYFSDLNFQAPMVVT	51	65	38.710	2
20	YFSDLNFQAPMVVTS	52	66	30.700	1

21	FSDLNFQAPMVVTSS	53	67	26.210	1
22	SDLNFQAPMVVTSST	54	68	42.010	1
23	SENQYFQSAIWSGFI	84	98	47.890	1
24	ENQYFQSAIWSGFIK	85	99	45.234	7
25	NQYFQSAIWSGFIKV	86	100	33.971	10
26	QYFQSAIWSGFIKVK	87	101	26.909	7
27	YFQSAIWSGFIKVKK	88	102	26.656	7
28	FQSAIWSGFIKVKKS	89	103	31.860	6
29	QSAIWSGFIKVKKSD	90	104	39.527	3
30	KKSDEYTFATSADNH	101	115	47.130	1
31	KSDEYTFATSADNHV	102	116	28.460	1
32	SDEYTFATSADNHVT	103	117	26.300	1
33	DEYTFATSADNHVTM	104	118	28.540	1
34	EYTFATSADNHVTMW	105	119	24.570	1
35	ASNSNKIRLEKGRLY	129	143	39.597	3
36	SNSNKIRLEKGRLYQ	130	144	31.510	5
37	NSNKIRLEKGRLYQI	131	145	33.454	10
38	SNKIRLEKGRLYQIK	132	146	32.633	12
39	NKIRLEKGRLYQIKI	133	147	33.608	10
40	KIRLEKGRLYQIKIQ	134	148	30.582	5
41	KKEVISSDNLQLPEL	171	185	44.250	1
42	TVDVKNKRTFLSPWI	222	236	43.800	1
43	VDVKNKRTFLSPWIS	223	237	45.360	1
44	KNKRTFLSPWISNIH	226	240	46.530	1
45	EARHPLVAAYPIVHV	279	293	35.393	3
46	ARHPLVAAYPIVHVD	280	294	27.805	2
47	RHPLVAAYPIVHVDM	281	295	33.825	4
48	HPLVAAYPIVHVDME	282	296	37.080	2
49	VHASFFDIGGSVSAG	338	352	36.270	1
50	HASFFDIGGSVSAGF	339	353	21.140	1
51	ASFFDIGGSVSAGFS	340	354	20.300	1
52	SFFDIGGSVSAGFSN	341	355	30.490	1
53	VSAGFSNSNSSTVAI	349	363	46.440	1
54	SNSSTVAIDHSLSLA	356	370	36.490	1
55	NSSTVAIDHSLSLAG	357	371	35.970	1
56	SSTVAIDHSLSLAGE	358	372	39.260	1
57	STVAIDHSLSLAGER	359	373	43.720	1
58	NTADTARLNANIRYV	382	396	37.920	1
59	TADTARLNANIRYVN	383	397	32.790	1
60	ADTARLNANIRYVNT	384	398	33.150	1
61	DTARLNANIRYVNTG	385	399	41.540	1
62	LNANIRYVNTGTAPI	389	403	34.090	2
63	NANIRYVNTGTAPIY	390	404	33.075	6
64	ANIRYVNTGTAPIYN	391	405	30.664	5
65	NIRYVNTGTAPIYNV	392	406	34.751	7
66	IRYVNTGTAPIYNVL	393	407	31.298	5

67	RYVNTGTAPIYNVLP	394	408	31.470	2
68	TGTAPIYNVLPTTSL	398	412	19.880	2
69	GTAPIYNVLPTTSLV	399	413	17.483	3
70	TAPIYNVLPTTSLVL	400	414	23.045	6
71	APIYNVLPTTSLVLG	401	415	18.888	5
72	PIYNVLPTTSLVLGK	402	416	25.030	6
73	IYNVLPTTSLVLGKN	403	417	25.940	5
74	YNVLPTTSLVLGKNQ	404	418	17.355	2
75	NVLPTTSLVLGKNQT	405	419	19.070	1
76	LPTTSLVLGKNQTLA	407	421	31.695	2
77	PTTSLVLGKNQTLAT	408	422	34.118	4
78	TTSLVLGKNQTLATI	409	423	24.633	4
79	TSLVLGKNQTLATIK	410	424	25.523	3
80	SLVLGKNQTLATIKA	411	425	33.870	2
81	LATIKAKENQLSQIL	420	434	43.460	1
82	KENQLSQILAPNNYY	426	440	41.670	1
83	DFSSTPITMNYNQFL	455	469	26.580	1
84	FSSTPITMNYNQFLE	456	470	20.080	1
85	SSTPITMNYNQFLEL	457	471	28.214	8
86	STPITMNYNQFLELE	458	472	24.688	9
87	TPITMNYNQFLELEK	459	473	22.102	10
88	PITMNYNQFLELEKT	460	474	23.072	10
89	ITMNYNQFLELEKTK	461	475	21.445	8
90	TMNYNQFLELEKTKQ	462	476	31.652	6
91	NYNQFLELEKTKQLR	464	478	42.550	2
92	YNQFLELEKTKQLRL	465	479	35.883	3
93	NQFLELEKTKQLRLD	466	480	46.460	2
94	EKTKQLRLDTDQVYG	472	486	34.810	1
95	KTKQLRLDTDQVYGN	473	487	34.740	1
96	TKQLRLDTDQVYGNI	474	488	26.950	1
97	KQLRLDTDQVYGNIA	475	489	28.630	1
98	SEVLPQIQETTARII	507	521	16.360	1
99	EVLPQIQETTARIIF	508	522	12.430	1
100	VLPQIQETTARIIFN	509	523	30.645	2
101	LPQIQETTARIIFNG	510	524	18.930	1
102	PQIQETTARIIFNGK	511	525	27.730	1
103	QIQETTARIIFNGKD	512	526	28.360	1
104	RIIFNGKDLNLVERR	519	533	49.390	2
105	IFNGKDLNLVERRIA	521	535	33.795	2
106	FNGKDLNLVERRIAA	522	536	29.137	9
107	NGKDLNLVERRIAAV	523	537	23.106	10
108	GKDLNLVERRIAAVN	524	538	24.347	10
109	KDLNLVERRIAAVNP	525	539	24.861	10
110	DLNLVERRIAAVNPS	526	540	36.144	8
111	EFDFNFDQQTSQNIK	578	592	48.220	1
112	FDFNFDQQTSQNIKN	579	593	33.890	1

113	DFNFDQQTSQNIKNQ	580	594	29.210	1
114	FNFDQQTSQNIKNQL	581	595	27.600	1
115	QTSQNIKNQLAELNA	586	600	46.640	1
116	TSQNIKNQLAELNAT	587	601	40.680	1
117	SQNIKNQLAELNATN	588	602	44.080	1
118	QNIKNQLAELNATNI	589	603	43.480	1
119	NIKNQLAELNATNIY	590	604	41.400	2
120	IKNQLAELNATNIYT	591	605	27.820	1
121	KNQLAELNATNIYTV	592	606	20.250	1
122	NQLAELNATNIYTVL	593	607	33.430	1
123	TNIYTVLDKIKLNAK	601	615	44.167	3
124	NIYTVLDKIKLNAKM	602	616	42.020	3
125	IYTVLDKIKLNAKMN	603	617	43.700	2
126	YTVLDKIKLNAKMNI	604	618	34.870	5
127	TVLDKIKLNAKMNIL	605	619	31.665	6
128	VLDKIKLNAKMNILI	606	620	30.858	9
129	LDKIKLNAKMNILIR	607	621	25.710	11
130	DKIKLNAKMNILIRD	608	622	31.852	12
131	KIKLNAKMNILIRDK	609	623	30.463	11
132	IKLNAKMNILIRDKR	610	624	34.458	5
133	KLNAKMNILIRDKRF	611	625	47.615	4
134	LNAKMNILIRDKRFH	612	626	33.812	5
135	NAKMNILIRDKRFHY	613	627	23.771	7
136	AKMNILIRDKRFHYD	614	628	24.246	7
137	KMNILIRDKRFHYDR	615	629	27.006	8
138	MNILIRDKRFHYDRN	616	630	26.503	6
139	NILIRDKRFHYDRNN	617	631	34.716	5
140	ILIRDKRFHYDRNNI	618	632	35.100	1
141	LIRDKRFHYDRNNIA	619	633	19.830	1
142	IRDKRFHYDRNNIAV	620	634	33.430	5
143	RDKRFHYDRNNIAVG	621	635	25.750	4
144	DKRFHYDRNNIAVGA	622	636	32.048	8
145	KRFHYDRNNIAVGAD	623	637	29.900	8
146	RFHYDRNNIAVGADE	624	638	19.310	2
147	STEGLLLNIDKDIRK	652	666	44.310	1
148	TEGLLLNIDKDIRKI	653	667	32.490	2
149	EGLLLNIDKDIRKIL	654	668	35.257	3
150	GLLLNIDKDIRKILS	655	669	34.335	2
151	LLLNIDKDIRKILSG	656	670	28.320	1
152	LLNIDKDIRKILSGY	657	671	43.120	1
153	LNIDKDIRKILSGYI	658	672	17.780	1
154	NIDKDIRKILSGYIV	659	673	28.537	6
155	IDKDIRKILSGYIVE	660	674	33.084	8
156	DKDIRKILSGYIVEI	661	675	32.668	11
157	KDIRKILSGYIVEIE	662	676	32.547	11
158	DIRKILSGYIVEIED	663	677	36.588	4

159	IRKILSGYIVEIEDT	664	678	47.900	1
160	VINDRYDMLNISSLR	684	698	30.325	2
161	INDRYDMLNISSLRQ	685	699	25.360	2
162	NDRYDMLNISSLRQD	686	700	31.887	3
163	DRYDMLNISSLRQDG	687	701	32.450	3
164	RYDMLNISSLRQDGK	688	702	32.453	4
165	YDMLNISSLRQDGKT	689	703	40.680	1
166	NISSLRQDGKTFIDF	693	707	34.220	1
167	ISSLRQDGKTFIDFK	694	708	29.620	1
168	SSLRQDGKTFIDFKK	695	709	41.190	1
169	NDKLPLYISNPNYKV	711	725	31.730	2
170	DKLPLYISNPNYKVN	712	726	31.070	4
171	KLPLYISNPNYKVNV	713	727	31.173	7
172	LPLYISNPNYKVNVY	714	728	23.040	5
173	PLYISNPNYKVNVYA	715	729	26.710	4
174	LYISNPNYKVNVYAV	716	730	23.085	2
175	TSTNGIKKILIFSKK	745	759	41.890	1
176	STNGIKKILIFSKKG	746	760	42.060	1
177	TNGIKKILIFSKKGY	747	761	30.093	7
178	NGIKKILIFSKKGYE	748	762	27.444	7
179	GIKKILIFSKKGYEI	749	763	28.234	9
180	IKKILIFSKKGYEIG	750	764	30.432	9

Table S5. Epitope sequences with multiple allele hits, presented in alphabetical order of the sequence. Start and End denote the location of the epitope along the PA amino acid sequence (Table S1).

N alleles	Peptide/Epitope	Start	End	Allele	ic50
1	ADTARLNANIRYVNT	384	398	DRB1*13:02	33.15
	AKMNILIRDKRFHYD	614	628	DRB1*03:01	22.74
	AKMNILIRDKRFHYD	614	628	DRB1*11:02	22.08
	AKMNILIRDKRFHYD	614	628	DRB1*11:03	15.96
	AKMNILIRDKRFHYD	614	628	DRB1*11:04	23.40
	AKMNILIRDKRFHYD	614	628	DRB1*13:01	22.08
	AKMNILIRDKRFHYD	614	628	DRB1*13:04	19.68
7	AKMNILIRDKRFHYD	614	628	DRB1*13:06	43.78
1	ALSTILVSSTGNLEV	12	26	DRB1*13:02	33.76
	ANIRYVNTGTAPIYN	391	405	DRB1*01:01	23.04
	ANIRYVNTGTAPIYN	391	405	DRB1*04:04	33.85
	ANIRYVNTGTAPIYN	391	405	DRB1*04:08	47.38
	ANIRYVNTGTAPIYN	391	405	DRB1*07:01	18.70
5	ANIRYVNTGTAPIYN	391	405	DRB1*10:01	30.35
	APIYNVLPTTSLVLG	401	415	DRB1*01:01	5.65

	APIYNVLPTTSLVLG	401	415	DRB1*07:01	10.73
	APIYNVLPTTSLVLG	401	415	DRB1*10:01	14.39
	APIYNVLPTTSLVLG	401	415	DRB1*16:01	41.65
5	APIYNVLPTTSLVLG	401	415	DRB1*16:02	22.02
	ARHPLVAAYPIVHVD	280	294	DRB1*01:01	31.28
2	ARHPLVAAYPIVHVD	280	294	DRB1*10:01	24.33
1	ASFFDIGGSVSAGFS	340	354	DRB1*01:01	20.30
	ASNSNKIRLEKGRLY	129	143	DRB1*11:02	40.57
	ASNSNKIRLEKGRLY	129	143	DRB1*13:01	40.57
3	ASNSNKIRLEKGRLY	129	143	DRB1*13:04	37.65
1	DEYTFATSADNHVTM	104	118	DRB1*10:01	28.54
1	DFNFDQQTSQNIKNQ	580	594	DRB1*01:01	29.21
1	DFSSTPITMNYNQFL	455	469	DRB1*13:02	26.58
	DIRKILSGYIVEIED	663	677	DRB1*01:01	24.05
	DIRKILSGYIVEIED	663	677	DRB1*01:02	48.08
	DIRKILSGYIVEIED	663	677	DRB1*07:01	33.36
4	DIRKILSGYIVEIED	663	677	DRB1*15:01	40.86
	DKDIRKILSGYIVEI	661	675	DRB1*01:01	11.29
	DKDIRKILSGYIVEI	661	675	DRB1*01:02	25.72
	DKDIRKILSGYIVEI	661	675	DRB1*07:01	13.40
	DKDIRKILSGYIVEI	661	675	DRB1*10:01	46.20
	DKDIRKILSGYIVEI	661	675	DRB1*11:02	40.12
	DKDIRKILSGYIVEI	661	675	DRB1*12:01	48.45
	DKDIRKILSGYIVEI	661	675	DRB1*13:01	40.12
	DKDIRKILSGYIVEI	661	675	DRB1*13:04	40.74
	DKDIRKILSGYIVEI	661	675	DRB1*15:01	16.88
	DKDIRKILSGYIVEI	661	675	DRB1*15:03	30.11
11	DKDIRKILSGYIVEI	661	675	DRB1*16:02	46.32
	DKIKLNAKMNILIRD	608	622	DRB1*01:01	49.02
	DKIKLNAKMNILIRD	608	622	DRB1*11:02	15.62
	DKIKLNAKMNILIRD	608	622	DRB1*11:03	27.75
	DKIKLNAKMNILIRD	608	622	DRB1*11:04	43.46
	DKIKLNAKMNILIRD	608	622	DRB1*13:01	15.62
	DKIKLNAKMNILIRD	608	622	DRB1*13:02	10.55
	DKIKLNAKMNILIRD	608	622	DRB1*13:04	23.44
	DKIKLNAKMNILIRD	608	622	DRB1*13:06	31.08
	DKIKLNAKMNILIRD	608	622	DRB1*14:01	41.98

	DKIKLNAKMNILIRD	608	622	DRB1*14:02	47.60
	DKIKLNAKMNILIRD	608	622	DRB1*14:06	34.12
12	DKIKLNAKMNILIRD	608	622	DRB1*14:54	41.98
	DKLPLYISNPNYKVN	712	726	DRB1*01:01	22.54
	DKLPLYISNPNYKVN	712	726	DRB1*13:02	15.17
	DKLPLYISNPNYKVN	712	726	DRB1*14:02	40.25
4	DKLPLYISNPNYKVN	712	726	DRB1*16:02	46.32
	DKRFHYDRNNIAVGA	622	636	DRB1*01:01	23.33
	DKRFHYDRNNIAVGA	622	636	DRB1*04:01	8.98
	DKRFHYDRNNIAVGA	622	636	DRB1*04:05	45.63
	DKRFHYDRNNIAVGA	622	636	DRB1*04:08	18.53
	DKRFHYDRNNIAVGA	622	636	DRB1*10:01	46.16
	DKRFHYDRNNIAVGA	622	636	DRB1*13:02	27.24
	DKRFHYDRNNIAVGA	622	636	DRB1*13:03	47.11
8	DKRFHYDRNNIAVGA	622	636	DRB1*14:02	39.40
	DLNLVERRIAAVNPS	526	540	DRB1*08:04	40.98
	DLNLVERRIAAVNPS	526	540	DRB1*11:01	36.12
	DLNLVERRIAAVNPS	526	540	DRB1*11:02	44.27
	DLNLVERRIAAVNPS	526	540	DRB1*11:03	27.08
	DLNLVERRIAAVNPS	526	540	DRB1*11:04	15.29
	DLNLVERRIAAVNPS	526	540	DRB1*13:01	44.27
	DLNLVERRIAAVNPS	526	540	DRB1*13:05	36.12
8	DLNLVERRIAAVNPS	526	540	DRB1*13:06	45.02
1	DTARLNANIRYVNTG	385	399	DRB1*13:02	41.54
	DRYDMLNISSLRQDG	687	701	DRB1*01:01	29.96
	DRYDMLNISSLRQDG	687	701	DRB1*04:04	34.10
3	DRYDMLNISSLRQDG	687	701	DRB1*10:01	33.29
	EARHPLVAAYPIVHV	279	293	DRB1*01:01	33.17
	EARHPLVAAYPIVHV	279	293	DRB1*09:01	47.82
3	EARHPLVAAYPIVHV	279	293	DRB1*10:01	25.19
1	EFDFNFDQQTSQNIK	578	592	DRB1*01:01	48.22
	EGLLLNIDKDIRKIL	654	668	DRB1*03:01	22.54
	EGLLLNIDKDIRKIL	654	668	DRB1*13:02	34.05
3	EGLLLNIDKDIRKIL	654	668	DRB1*13:04	49.18
1	EKTKQLRLDTDQVYG	472	486	DRB1*03:01	34.81
	ENQYFQSAIWSGFIK	85	99	DRB1*10:01	33.91

	ENQYFQSAIWSGFIK	85	99	DRB1*09:01	46.45
	ENQYFQSAIWSGFIK	85	99	DRB1*01:01	46.58
	ENQYFQSAIWSGFIK	85	99	DPB1*02:02	46.81
	ENQYFQSAIWSGFIK	85	99	DPB1*04:01	47.63
	ENQYFQSAIWSGFIK	85	99	DPB1*126:01	47.63
7	ENQYFQSAIWSGFIK	85	99	DPB1*23:01	47.63
1	EVLPQIQETTARIIF	508	522	DRB1*07:01	12.43
1	EYTFATSADNHVTMW	105	119	DRB1*10:01	24.57
1	FDFNFDQQTSQNIKN	579	593	DRB1*01:01	33.89
1	FNFDQQTSQNIKNQL	581	595	DRB1*01:01	27.60
	FNGKDLNLVERRIAA	522	536	DRB1*08:04	32.56
	FNGKDLNLVERRIAA	522	536	DRB1*11:01	22.93
	FNGKDLNLVERRIAA	522	536	DRB1*11:02	36.35
	FNGKDLNLVERRIAA	522	536	DRB1*11:03	18.91
	FNGKDLNLVERRIAA	522	536	DRB1*11:04	11.00
	FNGKDLNLVERRIAA	522	536	DRB1*13:01	36.35
	FNGKDLNLVERRIAA	522	536	DRB1*13:04	45.73
	FNGKDLNLVERRIAA	522	536	DRB1*13:05	22.93
9	FNGKDLNLVERRIAA	522	536	DRB1*13:06	35.47
	FQSAIWSGFIKVKKS	89	103	DPB1*02:01	21.82
	FQSAIWSGFIKVKKS	89	103	DPB1*02:02	22.87
	FQSAIWSGFIKVKKS	89	103	DPB1*04:01	41.55
	FQSAIWSGFIKVKKS	89	103	DPB1*126:01	41.55
	FQSAIWSGFIKVKKS	89	103	DPB1*23:01	41.55
	FQSAIWSGFIKVKKS	89	103	DPB1*46:01	21.82
7	FSDLNFQAPMVVTSS	53	67	DRB1*01:01	26.21
1	FSSTPITMNYNQFLE	456	470	DRB1*13:02	20.08
	GIKKILIFSKKGYEI	749	763	DRB1*11:01	48.65
	GIKKILIFSKKGYEI	749	763	DRB1*11:02	19.86
	GIKKILIFSKKGYEI	749	763	DRB1*11:03	16.35
	GIKKILIFSKKGYEI	749	763	DRB1*11:04	15.88
	GIKKILIFSKKGYEI	749	763	DRB1*13:01	19.86
	GIKKILIFSKKGYEI	749	763	DRB1*13:04	16.91
	GIKKILIFSKKGYEI	749	763	DRB1*13:05	48.65
	GIKKILIFSKKGYEI	749	763	DRB1*13:06	28.27
9	GIKKILIFSKKGYEI	749	763	DRB1*14:06	39.68
	GKDLNLVERRIAAVN	524	538	DRB1*08:04	25.28

	GKDLNLVERRIAAVN	524	538	DRB1*11:01	17.79
	GKDLNLVERRIAAVN	524	538	DRB1*11:02	24.54
	GKDLNLVERRIAAVN	524	538	DRB1*11:03	14.55
	GKDLNLVERRIAAVN	524	538	DRB1*11:04	9.57
	GKDLNLVERRIAAVN	524	538	DRB1*13:01	24.54
	GKDLNLVERRIAAVN	524	538	DRB1*13:04	38.54
	GKDLNLVERRIAAVN	524	538	DRB1*13:05	17.79
	GKDLNLVERRIAAVN	524	538	DRB1*13:06	26.88
10	GKDLNLVERRIAAVN	524	538	DRB1*14:06	43.99
	GLLGYYFSDLNFQAP	47	61	DRB1*04:01	18.01
2	GLLGYYFSDLNFQAP	47	61	DRB1*04:08	49.13
	GLLLNIDKDIRKILS	655	669	DRB1*03:01	21.11
2	GLLLNIDKDIRKILS	655	669	DRB1*13:02	47.56
	GTAPIYNVLPTTSLV	399	413	DRB1*01:01	6.96
	GYYFSDLNFQAPMVV	50	64	DRB1*04:01	17.06
	GYYFSDLNFQAPMVV	50	64	DRB1*04:08	46.29
	GTAPIYNVLPTTSLV	399	413	DRB1*10:01	14.40
5	GTAPIYNVLPTTSLV	399	413	DRB1*16:02	31.09
1	HASFFDIGGSVSAGF	339	353	DRB1*01:01	21.14
	HPLVAAYPIVHVDME	282	296	DRB1*01:01	43.26
2	HPLVAAYPIVHVDME	282	296	DRB1*10:01	30.90
	IDKDIRKILSGYIVE	660	674	DRB1*01:01	14.08
	IDKDIRKILSGYIVE	660	674	DRB1*01:02	34.01
	IDKDIRKILSGYIVE	660	674	DRB1*07:01	15.23
	IDKDIRKILSGYIVE	660	674	DRB1*11:02	47.85
	IDKDIRKILSGYIVE	660	674	DRB1*13:01	47.85
	IDKDIRKILSGYIVE	660	674	DRB1*13:04	46.53
	IDKDIRKILSGYIVE	660	674	DRB1*15:01	21.20
8	IDKDIRKILSGYIVE	660	674	DRB1*15:03	37.92
	IFNGKDLNLVERRIA	521	535	DRB1*11:03	45.03
2	IFNGKDLNLVERRIA	521	535	DRB1*11:04	22.56
	IKKILIFSKKGYEIG	750	764	DRB1*11:01	49.05
	IKKILIFSKKGYEIG	750	764	DRB1*11:02	21.87
	IKKILIFSKKGYEIG	750	764	DRB1*11:03	16.19
	IKKILIFSKKGYEIG	750	764	DRB1*11:04	15.57
	IKKILIFSKKGYEIG	750	764	DRB1*13:01	21.87
	IKKILIFSKKGYEIG	750	764	DRB1*13:04	18.42
	IKKILIFSKKGYEIG	750	764	DRB1*13:05	49.05

	IKKILIFSKKGYEIG	750	764	DRB1*13:06	33.09
9	IKKILIFSKKGYEIG	750	764	DRB1*14:06	48.78
	IKLNAKMNILIRDKR	610	624	DRB1*11:02	31.08
	IKLNAKMNILIRDKR	610	624	DRB1*11:03	44.09
	IKLNAKMNILIRDKR	610	624	DRB1*13:01	31.08
	IKLNAKMNILIRDKR	610	624	DRB1*13:02	26.02
5	IKLNAKMNILIRDKR	610	624	DRB1*13:04	40.02
1	IKNQLAELNATNIYT	591	605	DRB1*01:01	27.82
1	ILIRDKRFHYDRNNI	618	632	DRB1*03:01	35.10
	INDRYDMLNISSLRQ	685	699	DRB1*01:01	23.11
2	INDRYDMLNISSLRQ	685	699	DRB1*10:01	27.61
1	IPLMALSTILVSSTG	8	22	DRB1*01:01	22.33
	IRDKRFHYDRNNIAV	620	634	DRB1*01:01	42.26
	IRDKRFHYDRNNIAV	620	634	DRB1*04:01	9.62
	IRDKRFHYDRNNIAV	620	634	DRB1*04:05	48.94
	IRDKRFHYDRNNIAV	620	634	DRB1*04:08	23.95
5	IRDKRFHYDRNNIAV	620	634	DRB1*13:02	42.38
1	IRYVNTGTAPIYNVL	393	407	DRB1*01:01	19.94
1	IRKILSGYIVEIEDT	664	678	DRB1*01:01	47.90
	IRYVNTGTAPIYNVL	393	407	DRB1*07:01	13.52
	IRYVNTGTAPIYNVL	393	407	DRB1*09:01	41.89
	IRYVNTGTAPIYNVL	393	407	DRB1*10:01	32.73
4	IRYVNTGTAPIYNVL	393	407	DRB1*16:02	48.41
	ITMNYNQFLELEKTK	461	475	DPB1*02:01	18.82
	ITMNYNQFLELEKTK	461	475	DPB1*02:02	15.95
	ITMNYNQFLELEKTK	461	475	DPB1*04:01	14.70
	ITMNYNQFLELEKTK	461	475	DPB1*126:01	14.70
	ITMNYNQFLELEKTK	461	475	DPB1*15:01	33.30
	ITMNYNQFLELEKTK	461	475	DPB1*23:01	14.70
	ITMNYNQFLELEKTK	461	475	DPB1*40:01	40.57
8	ITMNYNQFLELEKTK	461	475	DPB1*46:01	18.82
1	IYNVLPTTSLVLGKN	403	417	DRB1*01:01	8.47
1	ISSLRQDGKTFIDFK	694	708	DRB1*03:01	29.62

	IYNVLPTTSLVLGKN	403	417	DRB1*07:01	9.47
	IYNVLPTTSLVLGKN	403	417	DRB1*09:01	49.68
	IYNVLPTTSLVLGKN	403	417	DRB1*10:01	24.57
4	IYNVLPTTSLVLGKN	403	417	DRB1*16:02	37.51
	IYTVLDKIKLNAKMN	603	617	DRB1*11:03	46.85
2	IYTVLDKIKLNAKMN	603	617	DRB1*11:04	40.55
	KDIRKILSGYIVEIE	662	676	DRB1*01:01	12.61
	KDIRKILSGYIVEIE	662	676	DRB1*01:02	24.27
	KDIRKILSGYIVEIE	662	676	DRB1*07:01	14.34
	KDIRKILSGYIVEIE	662	676	DRB1*10:01	45.16
	KDIRKILSGYIVEIE	662	676	DRB1*11:02	40.73
	KDIRKILSGYIVEIE	662	676	DRB1*12:01	46.19
	KDIRKILSGYIVEIE	662	676	DRB1*13:01	40.73
	KDIRKILSGYIVEIE	662	676	DRB1*13:04	40.46
	KDIRKILSGYIVEIE	662	676	DRB1*15:01	17.17
	KDIRKILSGYIVEIE	662	676	DRB1*15:03	27.71
11	KDIRKILSGYIVEIE	662	676	DRB1*16:02	48.65
	KDLNLVERRIAAVNP	525	539	DRB1*08:04	24.99
	KDLNLVERRIAAVNP	525	539	DRB1*11:01	17.60
	KDLNLVERRIAAVNP	525	539	DRB1*11:02	25.73
	KDLNLVERRIAAVNP	525	539	DRB1*11:03	14.94
	KDLNLVERRIAAVNP	525	539	DRB1*11:04	8.81
	KDLNLVERRIAAVNP	525	539	DRB1*13:01	25.73
	KDLNLVERRIAAVNP	525	539	DRB1*13:04	37.26
	KDLNLVERRIAAVNP	525	539	DRB1*13:05	17.60
	KDLNLVERRIAAVNP	525	539	DRB1*13:06	27.12
10	KDLNLVERRIAAVNP	525	539	DRB1*14:06	48.83
1	KENQLSQILAPNNYY	426	440	DRB1*10:01	41.67
	KIKLNAKMNILIRDK	609	623	DRB1*01:01	36.48
	KIKLNAKMNILIRDK	609	623	DRB1*11:02	16.25
	KIKLNAKMNILIRDK	609	623	DRB1*11:03	25.55
	KIKLNAKMNILIRDK	609	623	DRB1*11:04	42.42
	KIKLNAKMNILIRDK	609	623	DRB1*13:01	16.25
	KIKLNAKMNILIRDK	609	623	DRB1*13:02	11.40
	KIKLNAKMNILIRDK	609	623	DRB1*13:04	23.10
	KIKLNAKMNILIRDK	609	623	DRB1*13:06	30.36
	KIKLNAKMNILIRDK	609	623	DRB1*14:01	49.15
	KIKLNAKMNILIRDK	609	623	DRB1*14:06	34.98
11	KIKLNAKMNILIRDK	609	623	DRB1*14:54	49.15
	KIRLEKGRLYQIKIQ	134	148	DRB1*01:01	35.95

	KIRLEKGRLYQIKIQ	134	148	DRB1*11:02	24.78
	KIRLEKGRLYQIKIQ	134	148	DRB1*11:03	36.83
	KIRLEKGRLYQIKIQ	134	148	DRB1*13:01	24.78
5	KIRLEKGRLYQIKIQ	134	148	DRB1*13:04	30.57
1	KKEVISSDNLQLPEL	171	185	DRB1*13:02	44.25
	KKRKVLIPLMALSTI	2	16	DPB1*03:01	23.39
	KKRKVLIPLMALSTI	2	16	DPB1*124:01	23.39
	KKRKVLIPLMALSTI	2	16	DPB1*14:01	32.24
	KKRKVLIPLMALSTI	2	16	DPB1*20:01	24.91
	KKRKVLIPLMALSTI	2	16	DPB1*50:01	42.31
	KKRKVLIPLMALSTI	2	16	DPB1*92:01	23.39
	KKRKVLIPLMALSTI	2	16	DRB1*01:01	7.01
	KKRKVLIPLMALSTI	2	16	DRB1*01:02	15.77
	KKRKVLIPLMALSTI	2	16	DRB1*04:04	44.52
	KKRKVLIPLMALSTI	2	16	DRB1*10:01	13.53
	KKRKVLIPLMALSTI	2	16	DRB1*11:04	35.23
12	KKRKVLIPLMALSTI	2	16	DRB1*13:06	47.09
1	KKSDEYTFATSADNH	101	115	DRB1*10:01	47.13
	KLNAKMNILIRDKRF	611	625	DRB1*11:02	47.15
	KLNAKMNILIRDKRF	611	625	DRB1*11:03	47.72
	KLNAKMNILIRDKRF	611	625	DRB1*13:01	47.15
4	KLNAKMNILIRDKRF	611	625	DRB1*13:04	48.44
	KLPLYISNPNYKVNV	713	727	DRB1*01:01	15.06
	KLPLYISNPNYKVNV	713	727	DRB1*11:02	42.45
	KLPLYISNPNYKVNV	713	727	DRB1*13:01	42.45
	KLPLYISNPNYKVNV	713	727	DRB1*13:02	11.05
	KLPLYISNPNYKVNV	713	727	DRB1*14:02	27.93
	KLPLYISNPNYKVNV	713	727	DRB1*15:02	48.51
7	KLPLYISNPNYKVNV	713	727	DRB1*16:02	30.76
	KMNILIRDKRFHYDR	615	629	DRB1*03:01	22.37
	KMNILIRDKRFHYDR	615	629	DRB1*11:02	21.35
	KMNILIRDKRFHYDR	615	629	DRB1*11:03	14.87
	KMNILIRDKRFHYDR	615	629	DRB1*11:04	24.05
	KMNILIRDKRFHYDR	615	629	DRB1*13:01	21.35
	KMNILIRDKRFHYDR	615	629	DRB1*13:04	20.48
	KMNILIRDKRFHYDR	615	629	DRB1*13:06	42.73
8	KMNILIRDKRFHYDR	615	629	DRB1*14:06	48.85
1	KNKRTFLSPWISNIH	226	240	DRB1*10:01	46.53

1	KNQLAELNATNIYTV	592	606	DRB1*01:01	20.25
1	KQLRLDTDQVYGNIA	475	489	DRB1*03:01	28.63
	KRFHYDRNNIAVGAD	623	637	DRB1*01:01	21.60
	KRFHYDRNNIAVGAD	623	637	DRB1*04:01	7.57
	KRFHYDRNNIAVGAD	623	637	DRB1*04:05	41.17
	KRFHYDRNNIAVGAD	623	637	DRB1*04:08	16.02
	KRFHYDRNNIAVGAD	623	637	DRB1*10:01	44.05
	KRFHYDRNNIAVGAD	623	637	DRB1*13:02	25.78
	KRFHYDRNNIAVGAD	623	637	DRB1*13:03	43.79
8	KRFHYDRNNIAVGAD	623	637	DRB1*14:02	39.22
	KRKVLIPLMALSTIL	3	17	DPB1*03:01	39.17
	KRKVLIPLMALSTIL	3	17	DPB1*124:01	39.17
	KRKVLIPLMALSTIL	3	17	DPB1*20:01	38.76
	KRKVLIPLMALSTIL	3	17	DPB1*92:01	39.17
	KRKVLIPLMALSTIL	3	17	DRB1*01:01	6.49
	KRKVLIPLMALSTIL	3	17	DRB1*01:02	14.44
	KRKVLIPLMALSTIL	3	17	DRB1*04:04	39.61
	KRKVLIPLMALSTIL	3	17	DRB1*10:01	14.14
	KRKVLIPLMALSTIL	3	17	DRB1*11:04	39.80
	KRKVLIPLMALSTIL	3	17	DRB1*13:06	44.97
11	KRKVLIPLMALSTIL	3	17	DRB1*15:01	48.52
1	KSDEYTFATSADNHV	102	116	DRB1*10:01	28.46
1	KTKQLRLDTDQVYGN	473	487	DRB1*03:01	34.74
	KVLIPLMALSTILVS	5	19	DRB1*01:01	11.15
	KVLIPLMALSTILVS	5	19	DRB1*01:02	27.96
3	KVLIPLMALSTILVS	5	19	DRB1*10:01	27.43
1	LATIKAKENQLSQIL	420	434	DRB1*08:04	43.46
	LDKIKLNAKMNILIR	607	621	DRB1*11:02	14.55
	LDKIKLNAKMNILIR	607	621	DRB1*11:03	25.92
	LDKIKLNAKMNILIR	607	621	DRB1*11:04	36.13
	LDKIKLNAKMNILIR	607	621	DRB1*13:01	14.55
	LDKIKLNAKMNILIR	607	621	DRB1*13:02	9.21
	LDKIKLNAKMNILIR	607	621	DRB1*13:04	20.56
	LDKIKLNAKMNILIR	607	621	DRB1*13:06	25.01
	LDKIKLNAKMNILIR	607	621	DRB1*14:01	34.10
	LDKIKLNAKMNILIR	607	621	DRB1*14:02	38.99
	LDKIKLNAKMNILIR	607	621	DRB1*14:06	29.69
11	LDKIKLNAKMNILIR	607	621	DRB1*14:54	34.10

	LGYYFSDLNFQAPMV	49	63	DRB1*04:01	14.96
2	LGYYFSDLNFQAPMV	49	63	DRB1*04:08	31.74
1	LIPLMALSTILVSST	7	21	DRB1*01:01	21.83
1	LIRDKRFHYDRNNIA	619	633	DRB1*04:01	19.83
	LLGYYFSDLNFQAPM	48	62	DRB1*04:01	15.22
2	LLGYYFSDLNFQAPM	48	62	DRB1*04:08	36.57
1	LLLNIDKDIRKILSG	656	670	DRB1*03:01	28.32
1	LLNIDKDIRKILSGY	657	671	DRB1*03:01	43.12
	LNAKMNILIRDKRFH	612	626	DRB1*11:02	37.05
	LNAKMNILIRDKRFH	612	626	DRB1*11:03	25.15
	LNAKMNILIRDKRFH	612	626	DRB1*11:04	37.78
	LNAKMNILIRDKRFH	612	626	DRB1*13:01	37.05
5	LNAKMNILIRDKRFH	612	626	DRB1*13:04	32.03
	LNANIRYVNTGTAPI	389	403	DRB1*04:04	47.85
2	LNANIRYVNTGTAPI	389	403	DRB1*07:01	20.33
1	LNIDKDIRKILSGYI	658	672	DRB1*07:01	17.78
	LPLYISNPNYKVNVY	714	728	DRB1*01:01	12.69
	LPLYISNPNYKVNVY	714	728	DRB1*13:02	10.52
	LPLYISNPNYKVNVY	714	728	DRB1*14:02	26.27
	LPLYISNPNYKVNVY	714	728	DRB1*15:02	39.90
5	LPLYISNPNYKVNVY	714	728	DRB1*16:02	25.82
1	LPQIQETTARIIFNG	510	524	DRB1*07:01	18.93
	LPTTSLVLGKNQTLA	407	421	DRB1*01:01	18.51
2	LPTTSLVLGKNQTLA	407	421	DRB1*01:02	44.88
	LSTILVSSTGNLEVI	13	27	DRB1*07:01	48.04
2	LSTILVSSTGNLEVI	13	27	DRB1*13:02	25.31
	LYISNPNYKVNVYAV	716	730	DRB1*01:01	25.04
2	LYISNPNYKVNVYAV	716	730	DRB1*13:02	21.13
	MKKRKVLIPLMALST	1	15	DPB1*03:01	21.40
	MKKRKVLIPLMALST	1	15	DPB1*124:01	21.40
	MKKRKVLIPLMALST	1	15	DPB1*14:01	30.84

	MKKRKVLIPLMALST	1	15	DPB1*20:01	23.52
	MKKRKVLIPLMALST	1	15	DPB1*35:01	45.23
	MKKRKVLIPLMALST	1	15	DPB1*50:01	40.13
	MKKRKVLIPLMALST	1	15	DPB1*92:01	21.40
	MKKRKVLIPLMALST	1	15	DRB1*01:01	13.24
	MKKRKVLIPLMALST	1	15	DRB1*01:02	30.09
	MKKRKVLIPLMALST	1	15	DRB1*10:01	18.15
11	MKKRKVLIPLMALST	1	15	DRB1*11:04	47.56
	MNILIRDKRFHYDRN	616	630	DRB1*03:01	25.25
	MNILIRDKRFHYDRN	616	630	DRB1*11:02	25.96
	MNILIRDKRFHYDRN	616	630	DRB1*11:03	17.44
	MNILIRDKRFHYDRN	616	630	DRB1*11:04	37.29
	MNILIRDKRFHYDRN	616	630	DRB1*13:01	25.96
6	MNILIRDKRFHYDRN	616	630	DRB1*13:04	27.12
	NAKMNILIRDKRFHY	613	627	DRB1*11:03	14.10
	NAKMNILIRDKRFHY	613	627	DRB1*13:04	18.75
	NAKMNILIRDKRFHY	613	627	DRB1*11:02	20.54
	NAKMNILIRDKRFHY	613	627	DRB1*13:01	20.54
	NAKMNILIRDKRFHY	613	627	DRB1*11:04	22.69
	NAKMNILIRDKRFHY	613	627	DRB1*03:01	26.75
7	NAKMNILIRDKRFHY	613	627	DRB1*13:06	43.03
	NANIRYVNTGTAPIY	390	404	DRB1*01:01	25.36
	NANIRYVNTGTAPIY	390	404	DRB1*04:04	34.99
	NANIRYVNTGTAPIY	390	404	DRB1*04:08	46.77
	NANIRYVNTGTAPIY	390	404	DRB1*07:01	15.49
	NANIRYVNTGTAPIY	390	404	DRB1*09:01	43.63
6	NANIRYVNTGTAPIY	390	404	DRB1*10:01	32.21
	NDKLPLYISNPNYKV	711	725	DRB1*01:01	44.46
2	NDKLPLYISNPNYKV	711	725	DRB1*13:02	19.00
	NDRYDMLNISSLRQD	686	700	DRB1*01:01	27.68
	NDRYDMLNISSLRQD	686	700	DRB1*04:04	34.52
3	NDRYDMLNISSLRQD	686	700	DRB1*10:01	33.46
	NGIKKILIFSKKGYE	748	762	DRB1*11:02	24.32
	NGIKKILIFSKKGYE	748	762	DRB1*11:03	19.99
	NGIKKILIFSKKGYE	748	762	DRB1*11:04	21.96
	NGIKKILIFSKKGYE	748	762	DRB1*13:01	24.32
	NGIKKILIFSKKGYE	748	762	DRB1*13:04	22.40
	NGIKKILIFSKKGYE	748	762	DRB1*13:06	35.88
7	NGIKKILIFSKKGYE	748	762	DRB1*14:06	43.24

	NGKDLNLVERRIAAV	523	537	DRB1*08:04	23.54
	NGKDLNLVERRIAAV	523	537	DRB1*11:01	16.44
	NGKDLNLVERRIAAV	523	537	DRB1*11:02	23.78
	NGKDLNLVERRIAAV	523	537	DRB1*11:03	14.07
	NGKDLNLVERRIAAV	523	537	DRB1*11:04	8.73
	NGKDLNLVERRIAAV	523	537	DRB1*13:01	23.78
	NGKDLNLVERRIAAV	523	537	DRB1*13:04	34.42
	NGKDLNLVERRIAAV	523	537	DRB1*13:05	16.44
	NGKDLNLVERRIAAV	523	537	DRB1*13:06	25.01
10	NGKDLNLVERRIAAV	523	537	DRB1*14:06	44.85
	NIDKDIRKILSGYIV	659	673	DRB1*01:01	17.14
	NIDKDIRKILSGYIV	659	673	DRB1*01:02	37.51
	NIDKDIRKILSGYIV	659	673	DRB1*07:01	13.03
	NIDKDIRKILSGYIV	659	673	DRB1*13:04	43.89
	NIDKDIRKILSGYIV	659	673	DRB1*15:01	22.63
6	NIDKDIRKILSGYIV	659	673	DRB1*15:03	37.02
	NIKNQLAELNATNIY	590	604	DRB1*01:01	35.72
2	NIKNQLAELNATNIY	590	604	DRB1*10:01	47.08
	NILIRDKRFHYDRNN	617	631	DRB1*03:01	30.63
	NILIRDKRFHYDRNN	617	631	DRB1*11:02	39.52
	NILIRDKRFHYDRNN	617	631	DRB1*11:03	25.24
	NILIRDKRFHYDRNN	617	631	DRB1*13:01	39.52
5	NILIRDKRFHYDRNN	617	631	DRB1*13:04	38.67
	NIRYVNTGTAPIYNV	392	406	DRB1*01:01	18.05
	NIRYVNTGTAPIYNV	392	406	DRB1*04:08	47.70
	NIRYVNTGTAPIYNV	392	406	DRB1*07:01	15.11
	NIRYVNTGTAPIYNV	392	406	DRB1*09:01	44.16
	NIRYVNTGTAPIYNV	392	406	DRB1*10:01	26.91
	NIRYVNTGTAPIYNV	392	406	DRB1*13:02	49.48
7	NIRYVNTGTAPIYNV	392	406	DRB1*16:02	41.85
1	NISSLRQDGKTFIDF	693	707	DRB1*03:01	34.22
	NIYTVLDKIKLNAKM	602	616	DRB1*11:01	43.57
	NIYTVLDKIKLNAKM	602	616	DRB1*11:04	38.92
3	NIYTVLDKIKLNAKM	602	616	DRB1*13:05	43.57
	NKIRLEKGRLYQIKI	133	147	DRB1*01:01	25.16
	NKIRLEKGRLYQIKI	133	147	DRB1*11:02	18.11
	NKIRLEKGRLYQIKI	133	147	DRB1*11:03	27.88
	NKIRLEKGRLYQIKI	133	147	DRB1*11:04	46.54
	NKIRLEKGRLYQIKI	133	147	DRB1*13:01	18.11

	NKIRLEKGRLYQIKI	133	147	DRB1*13:04	22.70
	NKIRLEKGRLYQIKI	133	147	DRB1*13:06	41.74
	NKIRLEKGRLYQIKI	133	147	DRB1*14:06	40.65
	NKIRLEKGRLYQIKI	133	147	DRB1*15:01	45.47
10	NKIRLEKGRLYQIKI	133	147	DRB1*15:03	49.72
	NQFLELEKTKQLRLD	466	480	DRB1*11:01	46.46
2	NQFLELEKTKQLRLD	466	480	DRB1*13:05	46.46
1	NQLAELNATNIYTVL	593	607	DRB1*01:01	33.43
	NQYFQSAIWSGFIKV	86	100	DPB1*02:01	25.94
	NQYFQSAIWSGFIKV	86	100	DPB1*02:02	24.10
	NQYFQSAIWSGFIKV	86	100	DPB1*04:01	27.24
	NQYFQSAIWSGFIKV	86	100	DPB1*126:01	27.24
	NQYFQSAIWSGFIKV	86	100	DPB1*15:01	47.80
	NQYFQSAIWSGFIKV	86	100	DPB1*23:01	27.24
	NQYFQSAIWSGFIKV	86	100	DPB1*46:01	25.94
	NQYFQSAIWSGFIKV	86	100	DRB1*01:01	44.75
	NQYFQSAIWSGFIKV	86	100	DRB1*09:01	48.25
10	NQYFQSAIWSGFIKV	86	100	DRB1*10:01	41.21
	NSNKIRLEKGRLYQI	131	145	DRB1*01:01	26.46
	NSNKIRLEKGRLYQI	131	145	DRB1*11:02	18.39
	NSNKIRLEKGRLYQI	131	145	DRB1*11:03	29.00
	NSNKIRLEKGRLYQI	131	145	DRB1*11:04	44.69
	NSNKIRLEKGRLYQI	131	145	DRB1*13:01	18.39
	NSNKIRLEKGRLYQI	131	145	DRB1*13:02	44.71
	NSNKIRLEKGRLYQI	131	145	DRB1*13:04	22.37
	NSNKIRLEKGRLYQI	131	145	DRB1*13:06	41.04
	NSNKIRLEKGRLYQI	131	145	DRB1*14:06	42.89
10	NSNKIRLEKGRLYQI	131	145	DRB1*15:01	46.60
1	NSSTVAIDHSLSLAG	357	371	DRB1*03:01	35.97
1	NTADTARLNANIRYV	382	396	DRB1*13:02	37.92
1	NVLPTTSLVLGKNQT	405	419	DRB1*07:01	19.07
	NYNQFLELEKTKQLR	464	478	DRB1*11:01	42.55
2	NYNQFLELEKTKQLR	464	478	DRB1*13:05	42.55
	PITMNYNQFLELEKT	460	474	DPB1*01:01	46.59
	PITMNYNQFLELEKT	460	474	DPB1*02:01	17.40
	PITMNYNQFLELEKT	460	474	DPB1*02:02	14.97
	PITMNYNQFLELEKT	460	474	DPB1*04:01	13.77

	PITMNYNQFLELEKT	460	474	DPB1*126:01	13.77
	PITMNYNQFLELEKT	460	474	DPB1*15:01	26.70
	PITMNYNQFLELEKT	460	474	DPB1*23:01	13.77
	PITMNYNQFLELEKT	460	474	DPB1*40:01	37.11
	PITMNYNQFLELEKT	460	474	DPB1*46:01	17.40
10	PITMNYNQFLELEKT	460	474	DRB1*13:02	29.24
	PIYNVLPTTSLVLGK	402	416	DRB1*01:01	6.12
	PIYNVLPTTSLVLGK	402	416	DRB1*07:01	8.85
	PIYNVLPTTSLVLGK	402	416	DRB1*09:01	45.35
	PIYNVLPTTSLVLGK	402	416	DRB1*10:01	16.65
	PIYNVLPTTSLVLGK	402	416	DRB1*16:01	48.23
6	PIYNVLPTTSLVLGK	402	416	DRB1*16:02	24.98
1	PLMALSTILVSSTGN	9	23	DRB1*01:01	26.85
	PLYISNPNYKVNVYA	715	729	DRB1*01:01	17.39
	PLYISNPNYKVNVYA	715	729	DRB1*13:02	14.70
	PLYISNPNYKVNVYA	715	729	DRB1*14:02	37.86
4	PLYISNPNYKVNVYA	715	729	DRB1*16:02	36.89
1	PQIQETTARIIFNGK	511	525	DRB1*07:01	27.73
	PTTSLVLGKNQTLAT	408	422	DRB1*01:01	14.25
	PTTSLVLGKNQTLAT	408	422	DRB1*01:02	36.04
	PTTSLVLGKNQTLAT	408	422	DRB1*13:02	39.67
4	PTTSLVLGKNQTLAT	408	422	DRB1*15:01	46.51
1	QGLLGYYFSDLNFQA	46	60	DRB1*04:01	47.70
1	QIQETTARIIFNGKD	512	526	DRB1*07:01	28.36
1	QNIKNQLAELNATNI	589	603	DRB1*10:01	43.48
	QSAIWSGFIKVKKSD	90	104	DPB1*02:01	39.69
	QSAIWSGFIKVKKSD	90	104	DPB1*02:02	39.20
3	QSAIWSGFIKVKKSD	90	104	DPB1*46:01	39.69
1	QTSQNIKNQLAELNA	586	600	DRB1*10:01	46.64
	QYFQSAIWSGFIKVK	87	101	DPB1*02:01	21.18
	QYFQSAIWSGFIKVK	87	101	DPB1*02:02	20.28
	QYFQSAIWSGFIKVK	87	101	DPB1*04:01	26.93
	QYFQSAIWSGFIKVK	87	101	DPB1*126:01	26.93
	QYFQSAIWSGFIKVK	87	101	DPB1*15:01	44.93
	QYFQSAIWSGFIKVK	87	101	DPB1*23:01	26.93

7	QYFQSAIWSGFIKVK	87	101	DPB1*46:01	21.18
	RDKRFHYDRNNIAVG	621	635	DRB1*01:01	32.59
	RDKRFHYDRNNIAVG	621	635	DRB1*04:01	9.77
	RDKRFHYDRNNIAVG	621	635	DRB1*04:08	22.50
4	RDKRFHYDRNNIAVG	621	635	DRB1*13:02	38.14
	RFHYDRNNIAVGADE	624	638	DRB1*04:01	10.38
2	RFHYDRNNIAVGADE	624	638	DRB1*04:08	28.24
	RHPLVAAYPIVHVDM	281	295	DRB1*01:01	24.69
	RHPLVAAYPIVHVDM	281	295	DRB1*09:01	49.96
	RHPLVAAYPIVHVDM	281	295	DRB1*10:01	19.69
4	RHPLVAAYPIVHVDM	281	295	DRB1*15:01	40.96
	RIIFNGKDLNLVERR	519	533	DPB1*02:01	49.39
2	RIIFNGKDLNLVERR	519	533	DPB1*46:01	49.39
	RKVLIPLMALSTILV	4	18	DRB1*01:01	6.60
	RKVLIPLMALSTILV	4	18	DRB1*01:02	14.97
	RKVLIPLMALSTILV	4	18	DRB1*04:04	39.04
	RKVLIPLMALSTILV	4	18	DRB1*10:01	15.83
	RKVLIPLMALSTILV	4	18	DRB1*13:06	47.53
6	RKVLIPLMALSTILV	4	18	DRB1*15:01	45.65
	RYDMLNISSLRQDGK	688	702	DRB1*01:01	29.09
	RYDMLNISSLRQDGK	688	702	DRB1*04:04	29.66
	RYDMLNISSLRQDGK	688	702	DRB1*10:01	30.97
4	RYDMLNISSLRQDGK	688	702	DRB1*11:04	40.09
	RYVNTGTAPIYNVLP	394	408	DRB1*01:01	41.42
2	RYVNTGTAPIYNVLP	394	408	DRB1*07:01	21.52
1	SDEYTFATSADNHVT	103	117	DRB1*10:01	26.30
1	SDLNFQAPMVVTSST	54	68	DRB1*01:01	42.01
1	SENQYFQSAIWSGFI	84	98	DRB1*10:01	47.89
1	SEVLPQIQETTARII	507	521	DRB1*07:01	16.36
1	SFFDIGGSVSAGFSN	341	355	DRB1*01:01	30.49
	SLVLGKNQTLATIKA	411	425	DRB1*01:01	28.62
2	SLVLGKNQTLATIKA	411	425	DRB1*13:02	39.12

	SNKIRLEKGRLYQIK	132	146	DRB1*01:01	22.34
	SNKIRLEKGRLYQIK	132	146	DRB1*01:02	46.76
	SNKIRLEKGRLYQIK	132	146	DRB1*11:02	16.65
	SNKIRLEKGRLYQIK	132	146	DRB1*11:03	25.29
	SNKIRLEKGRLYQIK	132	146	DRB1*11:04	39.03
	SNKIRLEKGRLYQIK	132	146	DRB1*13:01	16.65
	SNKIRLEKGRLYQIK	132	146	DRB1*13:02	41.41
	SNKIRLEKGRLYQIK	132	146	DRB1*13:04	21.03
	SNKIRLEKGRLYQIK	132	146	DRB1*13:06	35.96
	SNKIRLEKGRLYQIK	132	146	DRB1*14:06	36.21
	SNKIRLEKGRLYQIK	132	146	DRB1*15:01	40.77
12	SNKIRLEKGRLYQIK	132	146	DRB1*15:03	49.49
	SNSNKIRLEKGRLYQ	130	144	DRB1*01:01	40.21
	SNSNKIRLEKGRLYQ	130	144	DRB1*11:02	24.47
	SNSNKIRLEKGRLYQ	130	144	DRB1*11:03	38.79
	SNSNKIRLEKGRLYQ	130	144	DRB1*13:01	24.47
5	SNSNKIRLEKGRLYQ	130	144	DRB1*13:04	29.61
1	SNSSTVAIDHSLSLA	356	370	DRB1*03:01	36.49
1	SQNIKNQLAELNATN	588	602	DRB1*10:01	44.08
1	SSLRQDGKTFIDFKK	695	709	DRB1*03:01	41.19
	SSTPITMNYNQFLEL	457	471	DPB1*02:01	34.02
	SSTPITMNYNQFLEL	457	471	DPB1*02:02	27.82
	SSTPITMNYNQFLEL	457	471	DPB1*04:01	21.99
	SSTPITMNYNQFLEL	457	471	DPB1*126:01	21.99
	SSTPITMNYNQFLEL	457	471	DPB1*15:01	49.03
	SSTPITMNYNQFLEL	457	471	DPB1*23:01	21.99
	SSTPITMNYNQFLEL	457	471	DPB1*46:01	34.02
8	SSTPITMNYNQFLEL	457	471	DRB1*13:02	14.85
1	SSTVAIDHSLSLAGE	358	372	DRB1*03:01	39.26
1	STEGLLLNIDKDIRK	652	666	DRB1*13:02	44.31
1	STILVSSTGNLEVIQ	14	28	DRB1*13:02	29.30
1	STNGIKKILIFSKKG	746	760	DRB1*11:04	42.06
	STPITMNYNQFLELE	458	472	DPB1*02:01	24.96
	STPITMNYNQFLELE	458	472	DPB1*02:02	20.46
	STPITMNYNQFLELE	458	472	DPB1*04:01	16.53
	STPITMNYNQFLELE	458	472	DPB1*126:01	16.53

	STPITMNYNQFLELE	458	472	DPB1*15:01	36.24
	STPITMNYNQFLELE	458	472	DPB1*23:01	16.53
	STPITMNYNQFLELE	458	472	DPB1*40:01	49.46
	STPITMNYNQFLELE	458	472	DPB1*46:01	24.96
9	STPITMNYNQFLELE	458	472	DRB1*13:02	16.52
1	STVAIDHSLSLAGER	359	373	DRB1*03:01	43.72
1	TADTARLNANIRYVN	383	397	DRB1*13:02	32.79
	TAPIYNVLPTTSLVL	400	414	DRB1*01:01	5.44
	TAPIYNVLPTTSLVL	400	414	DRB1*04:04	48.64
	TAPIYNVLPTTSLVL	400	414	DRB1*07:01	11.50
	TAPIYNVLPTTSLVL	400	414	DRB1*10:01	12.14
	TAPIYNVLPTTSLVL	400	414	DRB1*16:01	39.69
6	TAPIYNVLPTTSLVL	400	414	DRB1*16:02	20.86
	TEGLLLNIDKDIRKI	653	667	DRB1*03:01	33.65
2	TEGLLLNIDKDIRKI	653	667	DRB1*13:02	31.33
	TGTAPIYNVLPTTSL	398	412	DRB1*01:01	16.30
2	TGTAPIYNVLPTTSL	398	412	DRB1*10:01	23.46
1	TILVSSTGNLEVIQA	15	29	DRB1*13:02	45.58
1	TKQLRLDTDQVYGNI	474	488	DRB1*03:01	26.95
	TMNYNQFLELEKTKQ	462	476	DPB1*02:01	34.65
	TMNYNQFLELEKTKQ	462	476	DPB1*02:02	29.11
	TMNYNQFLELEKTKQ	462	476	DPB1*04:01	30.50
	TMNYNQFLELEKTKQ	462	476	DPB1*126:01	30.50
	TMNYNQFLELEKTKQ	462	476	DPB1*23:01	30.50
6	TMNYNQFLELEKTKQ	462	476	DPB1*46:01	34.65
	TNGIKKILIFSKKGY	747	761	DRB1*11:02	25.14
	TNGIKKILIFSKKGY	747	761	DRB1*11:03	23.89
	TNGIKKILIFSKKGY	747	761	DRB1*11:04	26.21
	TNGIKKILIFSKKGY	747	761	DRB1*13:01	25.14
	TNGIKKILIFSKKGY	747	761	DRB1*13:04	24.71
	TNGIKKILIFSKKGY	747	761	DRB1*13:06	39.15
7	TNGIKKILIFSKKGY	747	761	DRB1*14:06	46.41
	TNIYTVLDKIKLNAK	601	615	DRB1*11:01	43.79
	TNIYTVLDKIKLNAK	601	615	DRB1*11:04	44.92
3	TNIYTVLDKIKLNAK	601	615	DRB1*13:05	43.79

	TPITMNYNQFLELEK	459	473	DPB1*01:01	48.59
	TPITMNYNQFLELEK	459	473	DPB1*02:01	17.61
	TPITMNYNQFLELEK	459	473	DPB1*02:02	15.36
	TPITMNYNQFLELEK	459	473	DPB1*04:01	13.20
	TPITMNYNQFLELEK	459	473	DPB1*126:01	13.20
	TPITMNYNQFLELEK	459	473	DPB1*15:01	26.96
	TPITMNYNQFLELEK	459	473	DPB1*23:01	13.20
	TPITMNYNQFLELEK	459	473	DPB1*40:01	36.26
	TPITMNYNQFLELEK	459	473	DPB1*46:01	17.61
10	TPITMNYNQFLELEK	459	473	DRB1*13:02	19.03
	TSLVLGKNQTLATIK	410	424	DRB1*01:01	15.39
	TSLVLGKNQTLATIK	410	424	DRB1*01:02	31.49
3	TSLVLGKNQTLATIK	410	424	DRB1*13:02	29.69
1	TSQNIKNQLAELNAT	587	601	DRB1*10:01	40.68
1	TSTNGIKKILIFSKK	745	759	DRB1*11:04	41.89
	TTSLVLGKNQTLATI	409	423	DRB1*01:01	11.83
	TTSLVLGKNQTLATI	409	423	DRB1*01:02	27.06
	TTSLVLGKNQTLATI	409	423	DRB1*13:02	26.14
4	TTSLVLGKNQTLATI	409	423	DRB1*15:01	33.50
1	TVDVKNKRTFLSPWI	222	236	DRB1*08:04	43.80
	TVLDKIKLNAKMNIL	605	619	DRB1*11:02	26.97
	TVLDKIKLNAKMNIL	605	619	DRB1*11:03	35.58
	TVLDKIKLNAKMNIL	605	619	DRB1*13:01	26.97
	TVLDKIKLNAKMNIL	605	619	DRB1*13:02	15.98
	TVLDKIKLNAKMNIL	605	619	DRB1*13:04	34.72
6	TVLDKIKLNAKMNIL	605	619	DRB1*14:06	49.77
1	VDVKNKRTFLSPWIS	223	237	DRB1*08:04	45.36
1	VHASFFDIGGSVSAG	338	352	DRB1*01:01	36.27
	VINDRYDMLNISSLR	684	698	DRB1*01:01	28.56
2	VINDRYDMLNISSLR	684	698	DRB1*10:01	32.09
	VLDKIKLNAKMNILI	606	620	DRB1*11:02	21.38
	VLDKIKLNAKMNILI	606	620	DRB1*11:03	38.75
	VLDKIKLNAKMNILI	606	620	DRB1*13:01	21.38
	VLDKIKLNAKMNILI	606	620	DRB1*13:02	10.81
	VLDKIKLNAKMNILI	606	620	DRB1*13:04	28.50
	VLDKIKLNAKMNILI	606	620	DRB1*13:06	39.11

	VLDKIKLNAKMNILI	606	620	DRB1*14:01	38.87
	VLDKIKLNAKMNILI	606	620	DRB1*14:06	40.05
9	VLDKIKLNAKMNILI	606	620	DRB1*14:54	38.87
	VLIPLMALSTILVSS	6	20	DRB1*01:01	17.01
2	VLIPLMALSTILVSS	6	20	DRB1*10:01	47.93
	VLPQIQETTARIIFN	509	523	DRB1*01:01	46.97
2	VLPQIQETTARIIFN	509	523	DRB1*07:01	14.32
1	VSAGFSNSNSSTVAI	349	363	DRB1*07:01	46.44
1	YDMLNISSLRQDGKT	689	703	DRB1*04:04	40.68
	YFQSAIWSGFIKVKK	88	102	DPB1*02:01	19.10
	YFQSAIWSGFIKVKK	88	102	DPB1*02:02	19.23
	YFQSAIWSGFIKVKK	88	102	DPB1*04:01	27.78
	YFQSAIWSGFIKVKK	88	102	DPB1*126:01	27.78
	YFQSAIWSGFIKVKK	88	102	DPB1*15:01	45.82
	YFQSAIWSGFIKVKK	88	102	DPB1*23:01	27.78
7	YFQSAIWSGFIKVKK	88	102	DPB1*46:01	19.10
1	YFSDLNFQAPMVVTS	52	66	DRB1*01:01	30.70
	YNQFLELEKTKQLRL	465	479	DRB1*01:01	45.61
	YNQFLELEKTKQLRL	465	479	DRB1*11:01	31.02
3	YNQFLELEKTKQLRL	465	479	DRB1*13:05	31.02
	YNVLPTTSLVLGKNQ	404	418	DRB1*01:01	23.78
2	YNVLPTTSLVLGKNQ	404	418	DRB1*07:01	10.93
	YTVLDKIKLNAKMNI	604	618	DRB1*11:02	36.01
	YTVLDKIKLNAKMNI	604	618	DRB1*11:03	36.03
	YTVLDKIKLNAKMNI	604	618	DRB1*13:01	36.01
	YTVLDKIKLNAKMNI	604	618	DRB1*13:02	24.82
5	YTVLDKIKLNAKMNI	604	618	DRB1*13:04	41.48
	YYFSDLNFQAPMVVT	51	65	DRB1*01:01	39.70
2	YYFSDLNFQAPMVVT	51	65	DRB1*04:01	37.72
